# Microbiome study in irradiated mice treated with BIO 300, a promising radiation countermeasure

**DOI:** 10.1186/s42523-021-00132-1

**Published:** 2021-10-09

**Authors:** Amrita K. Cheema, Yaoxiang Li, Jatinder Singh, Ryan Johnson, Michael Girgis, Stephen Y. Wise, Oluseyi O. Fatanmi, Michael D. Kaytor, Vijay K. Singh

**Affiliations:** 1 Department of Oncology, Lombardi Comprehensive Cancer Center, Georgetown University Medical Center, Washington, DC USA; 2 Department of Biochemistry, Molecular and Cellular Biology, Georgetown University Medical Center, Washington, DC USA; 3 Division of Radioprotectants, Department of Pharmacology and Molecular Therapeutics, F. Edward Hébert School of Medicine, Uniformed Services University of the Health Sciences, Bethesda, MD USA; 4 Armed Forces Radiobiology Research Institute, Uniformed Services University of the Health Sciences, Bethesda, MD USA; 5 Department of Preventive Medicine and Biostatistics, F. Edward Hébert School of Medicine, Uniformed Services University of the Health Sciences, Bethesda, MD USA; 6 Humanetics Corporation, Edina, MN 55435 USA

## Abstract

**Background:**

The mammalian gut harbors very complex and diverse microbiota that play an important role in intestinal homeostasis and host health. Exposure to radiation results in dysbiosis of the gut microbiota leading to detrimental pathophysiological changes to the host. To alleviate the effects of irradiation, several candidate countermeasures are under investigation. BIO 300, containing synthetic genistein formulated as an amorphous solid dispersion or as an aqueous suspension of nanoparticles, is a promising candidate under advanced development. The aim of this study was to investigate the effects of BIO 300 on the gut microbiome and metabolome of mice exposed to ^60^Co gamma-radiation. The gut microbiota and metabolome of control and drug-treated mice exposed to radiation was characterized by bacterial 16S rRNA amplicon sequencing and untargeted metabolomics.

**Results:**

We found that irradiation altered the *Firmicutes*/*Bacteroidetes* ratio and significantly decreased the relative abundance of *Lactobacillus*, both in BIO 300-treated and control mice; however, the ratio returned to near normal levels in BIO 300-treated mice by day 14 post-irradiation. Concomitantly, we also observed corrective shifts in metabolic pathways that were perturbed after irradiation.

**Conclusions:**

Overall, the data presented show that radiation exposure led to a relative depletion of commensals like *Lactobacillus* leading to an inflammatory metabolic phenotype while the majority of the drug-treated mice showed alleviation of this condition primarily by restoration of normal gut microbiota. These results indicate that the radioprotective effects of BIO 300, at least in part, may involve correction of the host-microbiome metabolic axis.

**Supplementary Information:**

The online version contains supplementary material available at 10.1186/s42523-021-00132-1.

## Background

The exposure of humans to high doses of ionizing radiation during a short period of time leads to acute radiation syndrome (ARS), and the type and degree of damage to the body is dependent on the dose. At lower doses (2–6 Gy), irradiation damages the hematopoietic system resulting in neutropenia and thrombocytopenia, leading to what is commonly known as hematopoietic-ARS (H-ARS) [1]. Moderately high levels of total-body irradiation (6–8 Gy) damage the gastrointestinal system, which manifests as diarrhea resulting in fluid loss, loss of mucosa, and gastrointestinal bleeding, and is known as gastrointestinal-ARS (GI-ARS) [2, 3]. At extremely high doses (> 8 Gy), cerebrovascular syndrome manifests and this condition is mostly fatal [4].

Gut microbiota plays a crucial role in intestinal homeostasis and provide several metabolic and immunoregulatory functions such as the production of vitamins, amino acids, short chain fatty acids and other metabolites, the biotransformation of bile, and the coevolution of the host’s immature immune system [5]. Short chain fatty acids are specifically important for gut homeostasis and health. This is due to their role as an energy source for gut epithelial cells, thereby improving the integrity of the host mucosal barrier and also offering immunomodulation and protection against pathogens in the gut [6]. These short-chain fatty acids have also been found to be potent stimuli for water and sodium absorption in the colon [7]. Human and murine gut microbiota share 90% similarity at the phyla level and comprise of mainly four bacterial phyla: *Firmicutes*, *Bacteriodetes*, *Proteobacteria* and *Actinobacteria* [8], with strict anaerobes outnumbering aerobes and facultative aerobes by up to 100-fold [9]. Stasis of gut microbiota is important and its dysbiosis has been implicated in diseases such as inflammatory bowel disease, metabolic disease, obesity, and cancer [10].

Irradiation was shown to result in significant alterations in the bacterial composition within large and small intestines in mice exposed to a single dose of 8 Gy ^60^Co γ-radiation [11]. It has also been demonstrated that in patients undergoing pelvic radiotherapy for gynecological cancer, the phyla *Firmicutes* and *Fusobacterium* change dramatically, with a 10% reduction in *Firmicutes* and a 3% increase of *Fusobacterium* [12]. Studies have disclosed that germ-free mice, which lack gut microbiota, are resistant to radiation-induced inflammation of the gut and other associated indications, such as diarrhea. This demonstrates that gut microbiota may influence or control intestinal inflammatory responses and/or disease development after intestinal damage from irradiation [13]. Recent studies in a murine model have illustrated that dysbiosis of gut microbiota post-irradiation stimulates susceptibility of the gut to inflammation [14]. In addition, the abundance of *Proteobacteria* and six other genera were inversely proportional to the levels of *Firmicutes*. Transplantation of this altered microbiota to germ-free, wild-type mice triggered the mice to become susceptible to dextran sodium sulfate-induced colitis and radiation injury. The authors of these studies concluded that modulation of interleukin-1β (IL-1β) signaling, in part by irradiation-altered microbiota, led to radiation-induced intestinal disease process [13, 14]. Wild-type mice irradiated with a low dose of radiation also showed changes in the gut microbiota, with a decrease in normal microbiota and an increase in opportunistic pathogens. These specific alterations in the microbiome manifested changes in the intestinal metabolome [15], with potential effects on physiological processes such as energy metabolism, cell-to-cell communication, and host immunity.

To mitigate the harmful effects of radiation exposure, the availability and administration of radiation countermeasures to protect individuals, prophylactically or therapeutically, is of utmost importance. Investigations are currently underway to identify and develop radiation countermeasures with beneficial effects during H-ARS and GI-ARS [16–18]. A promising radiation countermeasure under investigation, BIO 300 is a multimodal agent that is in advanced stages of development [19, 20]. The active pharmaceutical ingredient (API) in BIO 300 is synthetic genistein formulated as a nanoparticle suspension (BIO 300 oral suspension (OS)) or as an amorphous solid dispersion (BIO 300 oral powder (OP)). Genistein is a naturally occurring isoflavone found in soybeans. It has been shown to be an effective radiation countermeasure with radioprotective efficacy [19, 21–25]. Its mechanism of action is through its function as a selective agonist of the estrogen receptor-β (ERβ), which activates cell cycle checkpoints, represses cell growth, and inhibits inflammatory pathways [26]. Actively dividing cells are more radiosensitive than non-dividing cells and thus, genistein-mediated repression in the cellular growth rate may contribute to BIO 300’s radioprotective properties. This drug has been shown to arrest hematopoietic cells at the G2/M phase of cell cycle, resulting in a quiescent state and thereby reducing the harmful effects of irradiation [19]. Furthermore, genistein possesses antioxidant properties including the capability to scavenge reactive oxygen species (ROS), a product of radiolysis of water by irradiation, that lead to oxidative damage of DNA and other cellular components [19, 27, 28]. Importantly, it has been reported that oxidative stress actively stimulates the expansion of *Proteobacteria*, and it may also be detrimental to oxygen sensitive *Firmicutes* in the gut microbiota [29, 30].

Utilizing the mechanism of action of BIO 300 as a radiation countermeasure, we sought out to determine if BIO 300 may play a role in the maintenance of gut homeostasis. We hypothesized that the ability of BIO 300 to impart changes in the gut microbiota in a manner such that dysbiosis of gut microbiota, caused by radiation exposure, leads to restored homeostasis resulting in alleviation of radiation injury. In this study, we performed 16S rRNA amplicon sequencing and untargeted metabolomics in mouse fecal samples to describe changes in the fecal microbiota and metabolome as a surrogate marker for gut microbiota and metabolome of irradiated and BIO 300-treated mice. Our goal was to derive insights into the effects of this countermeasure as a prophylactic intervention towards stabilizing gut microbiota, as represented by a comparative analysis of the fecal microbiota and metabolome immediately prior to ^60^Co total-body radiation (9.2 Gy, 0.6 Gy/min) and intermittently over the course of 30 days post-exposure. We observed remarkable changes in the relative abundance of taxa post-irradiation in the drug-treated mice as compared to vehicle-treated mice. This was also accompanied by concomitant changes in metabolic pathways.

## Materials and methods

### Mice

Male CD2F1 mice, six to seven weeks old and specific pathogen free were purchased from Envigo (Dublin, VA, USA) and housed in the Uniformed Services University animal facility, an Association for Assessment and Accreditation of Laboratory Animal Care-International accredited facility as described earlier [31, 32]. All animal procedures were performed according to a protocol approved by Uniformed Services University Institutional Animal Care and Use Committee (IACUC). Research was conducted according to the *Guide for the Care and Use of Laboratory Animals* [33]. Moribundity was used as a surrogate for mortality, and euthanasia was used in order to minimize pain and distress to animals.

### Experimental design and drug administration

There were three groups of mice (16 mice per group) for this metagenomics and metabolomics study. There were four cages in each group with four mice in each cage. Group 1 received vehicle (0.5% Methocel A4M + 3% Kollidon 25) (termed ‘control’ throughout the text), and Groups 2 and 3 received either BIO 300 OS or BIO 300 OP (200 mg/kg/day) for six days prior to irradiation. BIO 300 OS is an aqueous liquid suspension of synthetic genistein nanoparticles with a median particle size of 200 nm. BIO 300 OP is an amorphous solid dispersion of genistein produced as a free-flowing dry powder with a median particle size of 160 µm. BIO 300 OP is prepared by hot-melt extrusion and milled to the final particle size. BIO 300 OP was slowly dispersed into vehicle and kept suspended with continuous stirring until dosing. Mice were administered their respective treatment twice per day *po* with 12 h between dosing. The drug/vehicle was administered using a 1 ml syringe and a 20-gauge feeding cannula, which had a ball tip. One sterile syringe and feeding needle was used for mice in each cage (four mice). Although the mice in each cage share the sipper tube in the water bottle and oral microorganisms as well, the feeding needle was wiped and disinfected between dosing on a gauze sponge moistened with 70% ethanol to reduce the microorganisms and saliva on the needle as an extra precaution. To avoid irritation to the mucosa, the feeding needle was wiped with a gauze sponge that was moistened with purified water. The volume of vehicle or drug administered was 200 µl/mouse.

### Irradiation

Mice were exposed to high dose ^60^Co γ-radiation at the Armed Forces Radiobiology Research Institute’s facility, as described previously [31]. After 6 days of vehicle or drug administration, mice were placed in compartmentalized and ventilated Plexiglas boxes and exposed to bilateral γ-irradiation (9.2 Gy, 0.6 Gy/min, approximately LD_70/30_ dose). After irradiation, mice were returned to their home cage and monitored for 30 days. Radiation dosimetry was based primarily on the alanine/EPR (Electron Paramagnetic Resonance) system [34], one of the most accurate methods currently accepted, and used for comparison between National Metrology institutions.

### Fecal pellet collection

As stated above, the drug or vehicle was administered for 6 days prior to irradiation. The day of irradiation was considered to be study day zero (SD0). Fecal pellets were collected from mice prior to drug or vehicle administration/irradiation in addition to post-treatment/post-irradiation, for a total of 5 time points (SD − 7, − 1, 3, 14, and 30 in relation to irradiation). For collecting feces samples, individual mice were placed in a sterile ventilated Plexiglas box. Using sterile forceps, two to three fecal pellets were collected in individually labeled sterile 1.5 ml Eppendorf tubes immediately after defecation. To prevent contamination between samples collected from different mice, the surface of the Plexiglas boxes and forceps were sterilized using 70% ethanol. The tubes containing the fecal pellets were immediately stored on dry ice before being transferred to − 80 °C until further analysis.

### Isolation of DNA from fecal pellets

Total DNA was isolated from the fecal pellets using QIAamp PowerFecal Pro DNA Kit (Qiagen, Germantown, MD, USA), and the procedure was accomplished according to the manufacturer’s guidelines. In brief, a single pellet was loaded into a PowerBead Pro tube containing solution CD1. The fecal samples were homogenized on a vortex mixer for 10 min at maximum speed and then centrifuged at 15,000×*g* for 1 min. To remove inhibitors, the supernatant was mixed with solution CD2, vortexed and centrifuged at 15,000×*g* for 1 min. To bind DNA, the resulting supernatant was mixed with solution CD3 and passed over an MB spin column. The column was then washed with solutions EA and C5, and bound DNA was eluted using 50 μl of solution C6. Finally, the concentration of DNA in the eluate was estimated using a Qubit™ 4 Fluorometer (Life Technologies, Carlsbad, CA, USA). The isolated DNA was stored at − 80 °C until further use.

### 16S rRNA sequencing library preparation

The amplicon library for the V3-V4 region of the 16S rRNA gene was prepared using QIAseq 16S/ITS Region Panel library construction kit (Qiagen, Germantown, MD, USA) as per the manufacturer’s directions. Briefly, the V3-V4 region of the 16S rRNA gene was PCR amplified in a 10 μl reaction volume containing UCP master mix, V3-V4 primers, and 1 ng of DNA. The following cycling conditions were used for PCR amplification: denature at 95 °C for 2 min, (95 °C 30 s, 50 °C 30 s, 72 °C 2 min) × 12 cycles, and final extension at 72 °C for 7 min. PCR reactions were set up in triplicate. After PCR was completed, the reactions were pooled and the amplified DNA was purified using QIAseq beads. Sample indices and sequencing adapters were added to the purified PCR product from the above step using dual indexed “phased primers” from Qiagen. The indexing PCR reaction contained purified PCR product from the previous step and UCP master mix in a total reaction volume of 50 μl. Cycling conditions for indexing PCR were: denature at 95 °C for 2 min, (95 °C 30 s, 60 °C 30 s, 72 °C 2 min) × 19 cycles, and a final extension at 72 °C 7 min. The amplified library from the above process was purified using QIAseq beads and eluted in 25 μl volume of nuclease free water. Size and quality of the indexed library was checked using Agilent DNA 1000 kit on an Agilent 2100 Bioanalyzer (Agilent Technologies, Inc., Santa Clara, CA, USA), and the library concentration was determined using the KAPA Library Quantification Kit for Illumina (KAPA Biosystems, Inc., Wilmington, MA, USA).

### Sequencing

The metagenomic library was prepared for sequencing on a MiSeq sequencing platform, as described in the MiSeq Reagent kit v3 Reagent Preparation Guide (Illumina, San Diego, CA, USA). Briefly, libraries were normalized to 4 nM and pooled volumetrically. The pooled sample library was denatured by mixing 5 μl of library and 5 μl of freshly prepared 0.2 N sodium hydroxide, vortexed and centrifuged at 250×*g* for 1 min. The tube was incubated for 5 min at room temperature and the reaction was stopped by adding 5 μl of Tris-Cl pH 7.0, mixed by vortexing, and centrifuged at 250×*g* for 1 min. Following this step, 985 μl of buffer HT1 was immediately added to the tube containing denatured library, and was then mixed and kept on ice. This process yielded a 20 pM library. The final concentration of the denatured library to be used for sequencing was determined based on the targeted cluster density. A MiSeq v3, 600 Cycle Reagent Cartridge (Illumina, San Diego, CA, USA) was used for sequencing. It was a paired end, dual indexed (151 Read I and 151 Read II, 8 Index Read 1, 8 Index Read 2) sequencing run.

### 16S rRNA sequence analysis

Raw 16S rRNA amplicons were converted to Amplicon Sequence Variants (ASVs) using the default DADA2 recommendations (version 1.18.0) [35]. In brief, the forward and reverse reads were trimmed to a length of 250 and 210, respectively. Additionally, reads were truncated to the first instance of a quality score less than or equal to 2. The maximum number of expected errors was set to 2 for the forward reads and 7 for the reverse reads. After assessing error rates, the reads were merged, chimera checked, and assigned taxonomic classification using the Silva Project’s 16S rRNA non-redundant reference database (version 132).

Reads were not rarefied [https://doi.org/10.1371/journal.pcbi.1003531], but were filtered out if they were associated with ASVs that were present in less than 5% of the samples. Furthermore, all reads classified as “uncharacterized” at the phylum level were removed from the study. Changes in microbial composition upon irradiation and/or drug treatment were inferred by performing comparative group analysis. Relative abundance data for each ASV was compared across samples via Bray–Curtis Dissimilarity matrix construction. Significant differences in Principal Component Analysis (PCoA) ordination clusters were computed using the analysis of variance of distance matrices test (Adonis) from within vegan R package. 999 permutations were performed for each Adonis test.

### Liquid chromatography–Mass Spectrometry (LC–MS) methods

Each fecal pellet sample was mixed with 600 µL of an extraction solution comprising of 10% (v/v) water, 30% (v/v) isopropanol, 20% (v/v) methanol, and 40% (v/v) chloroform. The extraction solution contained 0.1% debrisoquine (1 mg/ml in water) and 0.5% 4-Nitrobenzoic acid (1 mg/ml in methanol) as internal standards. The samples were homogenized on ice then kept at 4 °C for 15 min before being centrifuged at 15,493×*g* for 20 min at 4 °C. The supernatants were transferred to new tubes and a volume of 600 µl of chilled acetonitrile was added. Extracts were vortexed, and then kept at − 20 °C for 18 h. The samples were centrifuged as before at 15,493×*g* for 20 min at 4 °C and the supernatants were transferred to new tubes. Finally, the samples were vacuum dried, resuspended in 200 µl of 50/50 water/methanol, and then transferred to MS vials for LC–MS analysis.

A volume of 2 µl from each sample was injected onto a Waters Acquity BEH C18 1.7 μm, 2.1 × 50 mm column, which was kept at 40 °C for the metabolomics acquisition, and a Waters Acquity CSH C18 1.7 μm, 2.1 × 100 mm column, which was kept at 65 °C for the lipidomic acquisition utilizing an Acquity UPLC system coupled to a Xevo G2-S quadrupole-time-of-flight mass spectrometer with an electrospray ionization source (UPLC-ESI-QToF-MS—Ultra-performance liquid chromatography-lectrospray ionization-Quadrupole-time-of-flight) (Waters Corporation, Milford, MA). For the metabolomics profiling, the mobile phases consisted of 100% water with 0.1% formic acid (solvent A), acetonitrile containing 0.1% formic acid (solvent B), and 90:10 isopropanol/acetonitrile with 0.1% formic acid (solvent C). All solvents used were of LC–MS grade and were purchased from Fisher Scientific (Waltham, MA). The solvent flow rate was set to 0.4 ml/min with the column set at 40 °C. The LC gradient was as follows: Initial—95% A, 5% B; 0.5 min—95% A, 5% B; 8.0 min—2% A, 98% B; 9.0 min—11.8% B, 88.2% C; 10.5 min—11.2% B, 88.2% C; 11.5 min—50% A, 50% B; 12.5 min—95% A, 5% B; 13.0 min—95% A, 5%. The lipidomic solvents consisted of 50% water + 50% acetonitrile (ACN) + 0.1% formic acid + 10 mm ammonium formate (solvent A) and 90% isopropanol + 10% acetonitrile + 0.1% formic acid + 10 mm ammonium formate (solvent D). The flow rate was set to 0.45 ml/min and the LC gradient was as follows: Initial—60% A, 40% B; 0.5 min—60% A, 40% B; 8.0 min—0% A, 100% B; 8.5 min—0% A, 100.0% B; 9.0 min—60% A, 40% B; 11.0 min—60% A, 40% B.

The column eluent was introduced into the Xevo G2 mass spectrometer by electrospray operating in either negative or positive electrospray ionization mode. Positive mode had a capillary voltage of 3.00 kV and a sampling cone voltage of 30 V. Negative mode had a capillary voltage of 2.80 kV and had a sampling cone voltage of 30 V. The desolvation gas flow was set to 1000 L/hour and the desolvation temperature was set to 500 °C. The cone gas flow was 25 L/hour and the source temperature was set to 120 °C. The data was acquired in the sensitivity MS mode with a scan time of 0.300 s and an interscan time of 0.014 s. Accurate mass was maintained by infusing leucine enkephalin (556.2771 [M + H]^+^/554.2615 [M-H]^−^) in 50% aqueous acetonitrile (1.0 ng/ml) at a rate of 10 µl/min via the Lockspray interface every 10 s. The data was acquired in centroid mode from a mass range of 50 to 1200 m/z TOF–MS (Time-of-flight mass spectrometry) scanning. An aliquot of each sample was pooled and run as a quality control (QC) sample, which represented all metabolites present. The QC sample was run at the beginning of each acquisition to condition the column and then injected every 10 samples to ensure consistent retention times and intensities.

### Statistical analysis

The untargeted data was converted to NetCDF format using the Databridge tool in MassLynx (Waters Corporation, Milford, MA), and the XCMS [36] R package (version 3.12.0 URL: https://www.bioconductor.org/packages/release/bioc/html/xcms.html) was used for peak detection with ordered bijective interpolated warping algorithm utilized for retention time correction and parameters optimized using the Isotopologue Parameter Optimization (IPO) [37] R package (version 1.16.0 URL: https://bioconductor.org/packages/release/bioc/html/IPO.html). The mass to charge ratio and retention time (mz/rt) features were normalized to the internal standards debrisoquine and 4-nitrobenzoic acid present in the extraction solution in positive and negative modes respectively, then log transformed and pareto scaled. Unpaired t-tests were used to calculate up or down-regulation of each feature statistically, and significant metabolites were selected for MS/MS data acquisition and validation by the National Institute of Standards and Technology (NIST) 2017 database [38] fragmentation pattern matching. Kaplan Meier curves were constructed to view overall survival function throughout the study. Chi-square tests were performed to compare 30-day survival between the drug groups and the vehicle group. p-values of less than 0.05 were considered statistically significant and have been noted with an asterisk (*). The Wilcoxon-Mann–Whitney test was used to test the difference of Firmicutes/Bacteroidetes ratios between each group.

## Results

### Treatment with BIO 300 Oral Suspension or Oral Powder significantly increases survival

**Fig. 1:**
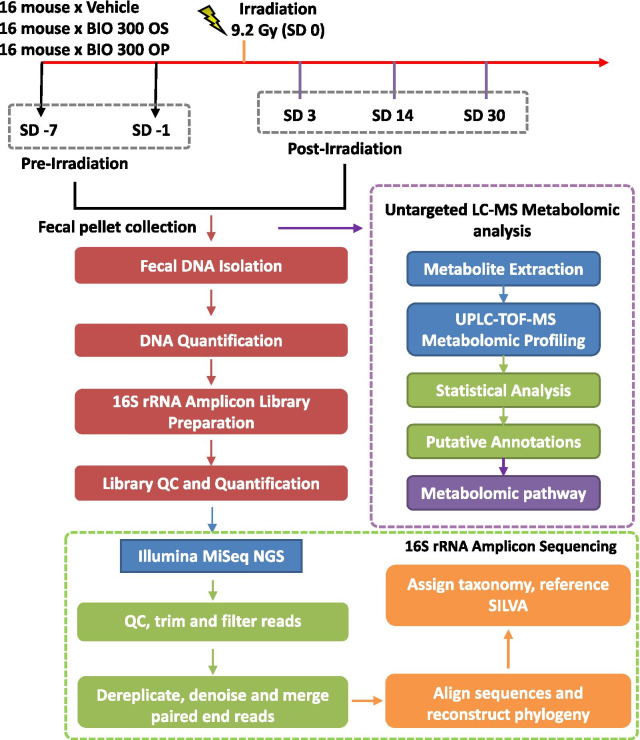
Experimental and analytical design of the study. Radioprotective efficacy, microbiome, and metabolomic studies of two different formulations of BIO 300, OS and OP, were conducted in irradiated CD2F1 male mice

To investigate the radioprotective efficacy of BIO 300 OS and BIO 300 OP, mice were administered a dose of 200 mg/kg *po* twice daily for six consecutive days prior to irradiation. Mice were exposed to 9.2 Gy (LD_70/30_) ^60^Co γ-radiation and survival was monitored for 30 days. Fecal samples were collected at 7 days (prior to the start of BIO 300 dosing) and 1 day prior to radiation exposure, as well as 3, 14, and 30-days post-irradiation. The samples were subjected to microbiome analysis as well as metabolomic/lipidomic analysis independently (Fig. 1).

**Fig. 2:**
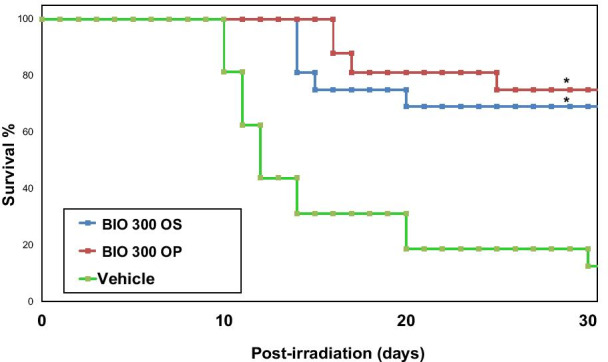
Radioprotective efficacy of BIO 300 formulations. Three groups of mice (n = 16) were administered either with BIO 300 OS, BIO 300 OP, or the vehicle *po* twice daily for six consecutive days prior to irradiation with 9.2 Gy. Survival was monitored for 30 days post-irradiation. *Indicates a significant difference compared to vehicle control (p < .05, log-rank test)

As expected, pre-treatment with BIO 300 emphatically enhanced survival, suggesting a strong protective effect against exposure to ionizing radiation. This was very consistent with earlier reports [19, 20, 39–41]. Treatment with BIO 300 OS resulted in a survival of 69% (11 survivors); mortality for this treatment group was observed between days 14–20. Mice administered BIO 300 OP had 12 mice remaining on day 30, representing a survival of 75% wherein mortality was observed between days 16–25. On the other hand, the vehicle-treated mouse group had only two survivors representing a 13% survival by day 30. While slightly different, both drug formulations accomplished a compelling increase in the survival rate compared to the vehicle group, as indicated by the Kaplan–Meier curve (Fig. 2).

### Administering BIO 300 restored microbiome composition back to normal in 14 days

We wanted to determine if some of the protective effects of BIO 300 leading to improved survival stemmed from restoration of post-irradiation dysbiosis in mice. We started by interrogating changes in fecal microbiome caused by irradiation and/or drug treatment using 16S rRNA amplicon sequencing; hence, we performed 16S rRNA amplicon sequencing on the mouse fecal samples to determine changes.

**Fig. 3:**
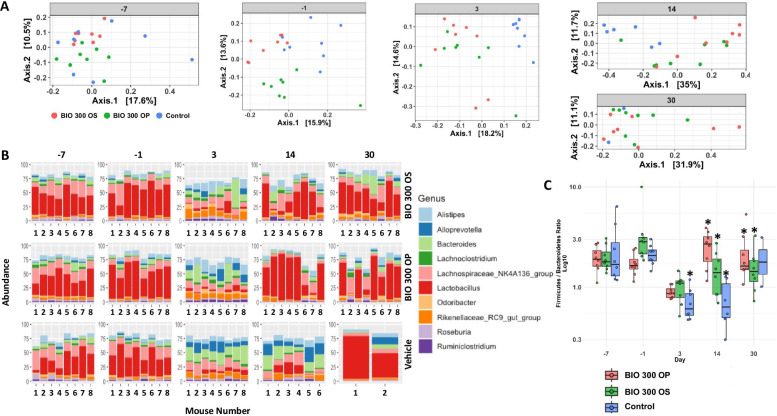
Alterations in radiation-induced microbiome diversity were alleviated by BIO 300 by day 14. Panel **A** Principal Coordinate Analysis showing clustering patterns for control mice and the treatment groups at 7 day and 1 day before irradiation as well as days 3, 14, and 30 post-irradiation. Panel **B** Relative abundance of the top 10 most abundant genera for the same cohort of mice across different treatment groups and time points. Panel **C** Log 10 Firmicutes/Bacteriodetes ratio over time between the three groups: BIO 300 OP, BIO 300 OS, and control

We used PCoA to visualize group differences (Fig. 3, Panel A) that showed that the three study groups (vehicle, BIO 300 OS, and BIO 300 OP) were clustered at day -7, while statistically significant differences were observed at day − 1 (Additional file 1: Table 1). A closer examination indicates that continuous dosing of BIO 300 increased the relative abundance of normal gut microflora like Lactobacillus (Fig. 3, Panel B). These results corroborate our earlier metabolomic and proteomic studies that show transient changes after drug treatment which are largely beneficial (PMID: 30870965). However, by the 3rd, 14th and 30th day, a clear separation was established between the vehicle-treated mice and the drug-treated groups, suggesting at least partial reversal of radiation-induced changes in gut microbiota.

Next, we examined the changes in the relative abundance at the genus level; patterns for the top 10 most abundant genera were visualized at pre and post-irradiation time points of SD -7, -3, 3, 14, and 30 in the vehicle, BIO 300 OS, and BIO 300 OP treated groups of mice (Fig. 3, Panel B). Radiation exposure resulted in a significant decrease in *Lactobacillus* and increase in *Bacteroides* and *Alloprevotella*.

At the phylum level, the mouse gut is comprised mainly of Bacteroidetes and Firmicutes. The ratio of Bacteroidetes/Firmicutes has always been measured to evaluate stability of the gut microbiome environment [42]. Treatment with oral BIO 300 did not appear to affect the Bacteroidetes/Firmicutes ratio. Exposure to radiation did impact the *Bacteroidetes*/*Firmicutes* ratio, however, BIO 300 was able to diminish these alterations by day 14 post-irradiation in a statistically significant manner (Fig. 3, Panel C).

BIO 300 treated mice (OS and OP) seemed to relatively mitigate dysbiosis by day 14 in part when compared to control mice. Furthermore, the control mice that survived appeared to have a comparative microflora composition, suggesting a strong correlation of restoration of gut dysbiosis with overall survival.

### Radiation-induced metabolic perturbations are corrected by oral BIO 300 after 14 days post-irradiation

**Fig. 4:**
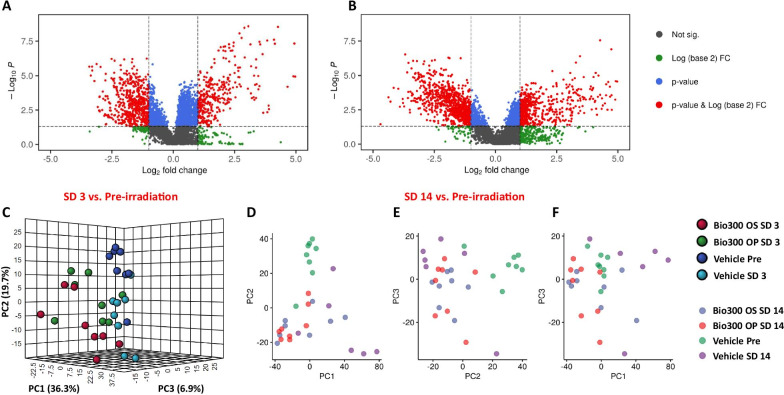
Multivariate analysis of metabolomics data showing clustering patterns between and across different treatment groups and pre- and post-irradiation time points. Panel **A** Volcano plot showing radiation effect on all detected features at SD3. Panel **B** Volcano plot illustrating radiation effect on all detected features at SD14. Panel **C** 3D Principal Component Analysis (3D-PCA) plot demonstrating the radiation effect on fecal detected features and the radioprotection of BIO 300 at SD3. Panel **D** 2D Principal Component Analysis (2D-PCA) PC 1 and PC 2 plot displaying the radiation effect on fecal detected features and the radioprotection of BIO 300 at SD14. Panel **E** 2D Principal Component Analysis (2D-PCA) PC 2 and PC 3 plot displaying the radiation effect on fecal detected features and the radioprotection of BIO 300 at SD14. Panel **F** 2D Principal Component Analysis (2D-PCA) PC 1 and PC 3 plot displaying the radiation effect on fecal detected features and the radioprotection of BIO 300 at SD14

Next, because changes in gut microbiota are likely to impact host metabolism, we hypothesized that irradiation of mice would result in alterations in the relative abundance of specific metabolites, some of which would be corrected by pre-treatment with BIO 300. Hence, we examined the global metabolomic and lipidomic profiles of the fecal extracts of the same cohort of mice at each time point using UPLC-ESI-QToF-MS. The data quality was controlled using the QC samples (Additional file 1: Figs. 1–8). All detected features were normalized based on the internal standards as well as the QC samples, and peak intensities were computed accordingly that resulted in 3614 and 3107 features in ESI positive and negative modes, respectively. Comparative analysis was performed by using normalized peak intensities for each feature within and across study samples. Volcano plots were used to visualize significantly dysregulated metabolites at days 3 and 14 compared to pre-irradiation (Fig. 4 panels A and B). The plots demonstrated that multiple metabolites were significantly altered (p < 0.05) after irradiation. Unsupervised clustering using principal component plot analyses between non-irradiated (vehicle pre-irradiation) and the irradiated mice (Fig. 4, panel C) are shown for vehicle or BIO 300 OS or OP formulation after 3 days post-irradiation. However, in alignment with our microbiome data, we observed little separation between the vehicle and BIO 300 groups at this time point showing minimal corrective effects of the drug. On the other hand, the 2D principal component analysis plots (Fig. 4, panel D, E and F) at day 14 showed a reasonable separation between mice receiving the drug as compared to the vehicle. Interestingly, mice receiving BIO 300 OP formulation showed a better metabolic corrective effect compared with mice receiving BIO 300 OS formulation, corroborating our earlier observations with the microbiome data.

**Fig. 5:**
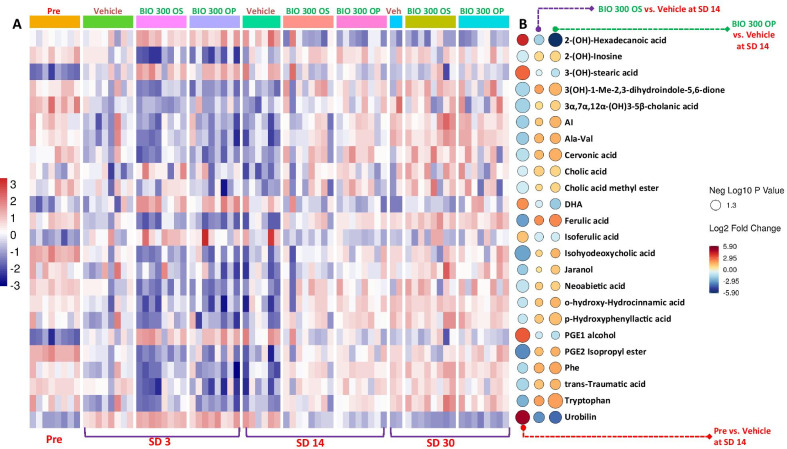
Prophylactic treatment with BIO 300 restores metabolic abundance dysregulated by irradiation. Panel **A** Heatmap for normalized intensities of annotated metabolites. Panel **B** Rain plot showing the recovering effects of both formulations of BIO 300 at SD14

Feature annotations based on fragmentation matching, their retention time, and their collision-induced dissociation (CID) fragments are listed in Additional file 1: Table 2. All annotated features with the corresponding p-values, fold change (FC), and false discovery rate (FDR) adjusted p-value are listed (Additional file 1: Table 3). Administration of either formulation of BIO 300 (OS or OP) showed a remarkable alleviation for all dysregulated metabolites, especially at SD14 as manifested by the pattern of metabolic changes (Fig. 5, panels A and B). This included different classes of metabolites including bile acids, phenolic acids, prostaglandins, indoles, and medium chain fatty acids (Fig. 5, Panels A and B).

**Fig. 6:**
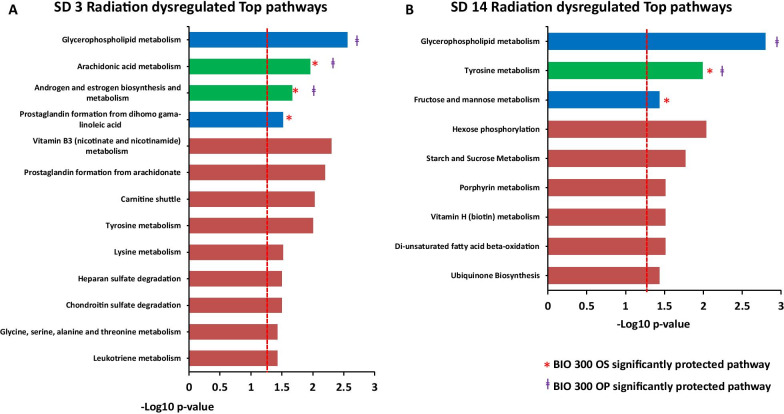
Pathway analysis showing significantly dysregulated pathway perturbations at SD3 (Panel **A**) and SD14 (Panel **B**). The bars in blue denote pathways corrected by BIO 300 OP, while bars in green are corrected by BIO 300 OS and OP

Furthermore, we performed pathway analysis with all detected features utilizing Mummichog v2.06. The outcome of Mummichog incorporates putative metabolite annotations and proposes involved pathways based on a statistical inference using built-in pathway information (Additional file 1: Table 4). Interestingly, several pathways were corrected by BIO 300 (both formulations) like arachidonic acid metabolism and androgen/estrogen biosynthesis and metabolism at SD3, indicating an early anti-inflammatory effect. Meanwhile, both formulations showed similar recovery profile over tryptophan metabolism pathway at SD14 (Fig. 6, panels OS and OP). Surprisingly, glycophospholipid metabolism was protected by BIO 300 OP at both time points (SD3 and SD14). Meanwhile, BIO 300 OS was able to revert prostaglandin synthesis pathway at SD3 and fructose/mannose metabolism pathway at SD14 (Additional file 1: Tables 5 and 6). The above results agree with our earlier metabolomics study using lung tissue from irradiated mice treated with BIO 300 [43].

## Discussion

In this study, we report findings from a murine model-based longitudinal study that was aimed at investigating radiation-induced changes in the microbiome and metabolome that were corrected at least in part by pre-treatment with BIO 300 either as a powder or suspension formulation. Mice were administered vehicle or BIO 300 for six days prior to irradiation (^60^Co γ-radiation, 9.2 Gy, 0.6 Gy/min). Fecal pellets were collected from mice pre-treated with either vehicle or drug at 7 (prior to BIO 300 treatment) and 1 day before radiation as well as on days 3, 14, and 30 after irradiation. The pellets were subject to 16S rRNA amplicon sequencing and untargeted metabolomics/lipidomics to infer a multi-omics narrative in a mouse model subjected to an acute high dose of γ-radiation. We found radiation-induced changes in the gut ecosystem that exhibited complex dynamics involving a decrease in the relative abundance of commensals like *Lactobacillus, Lachnospiraceae NK4A136 group, Bacteroides,* and *Alloprevotella*, and an increase in opportunistic pathogens like *Lachnoclostridium* that lead to an inflammatory phenotype by the induction of pro-inflammatory cytokines and the differentiation of Treg cells and microbial indicators of leaky gut such as *Alistipes* [44]. Subsequent analysis showed restoration of gut homeostasis in mice receiving either formulation of BIO 300 in a manner that was time dependent. For example, genus *Lactobacillus* showed a drastic decrease immediately post-irradiation and exhibited a very robust recovery profile by day 14. Interestingly, the survivors in the control group also showed a homeostasis in the gut commensals lending strong credence to the notion that the host-microbiome axis is crucial in modulating overall survival outcomes in response to radiation insults. Concomitant to these changes, we also observed an oscillatory shift in metabolite abundance that corroborated patterns observed with microbiome changes. We observed changes in the levels of an array of metabolites that are linked to microbial metabolism and directly impact host metabolism. Bile acids, phenolic acids like ferulic acid, medium chain fatty acids, and prostaglandins were among the most significant classes of metabolites that showed significant dysregulation upon acute exposure to γ-radiation and reverted back to near normal levels in mice that received BIO 300 by day 14. Studies have shown that ferulic acid, a hydroxycinnamic acid found in plants, is broken down by the esterase activity of *Lactobacillus* into 4-vinylguaiacol and hydroferulic acid; these bioactive metabolites have immense cerebro/neuro protective effects [45]. Strikingly, in our study we found a significant correlation in decrease of *Lactobacillus* after irradiation with a concomitant accumulation of ferulic acid while the levels reached near normal levels following correction of dysbiosis with BIO 300 treatment.

At the microbiome as well as the metabolite level, interestingly and somewhat unexpectedly, we found the oral powder formulation of BIO 300 seemed to exhibit better recovery effect than the oral suspension formulation. While this study is unable to determine the reason underlying this observation, on a practical level this is an easier route of administration and hence, is encouraging to follow up in future studies. The apparent enhanced recovery effect seen with BIO 300 OP dosing may be in part attributed to differences in the oral pharmacokinetics (e.g., C_max_ (maximum plasma concentration) or AUC (area under the curve)). The inability to detect statistically significant corrective changes 3 days post-irradiation may be linked to BIO 300’s mechanism of action. For example, the drug’s initial effect on cell cycle and DNA damage repair may mitigate a downstream inflammatory response, which if left untreated, would not present in a change in the microbiome at day 4 or later post-irradiation.

## Conclusion

In summary, these results show the potential of BIO 300 for reversing the adverse effects of radiation exposure on the gut microbiome and warrant further translational investigations. This is the first report demonstrating that the radiation countermeasure, BIO 300, imparts a radiation recovery benefit to animals in part by restoring gut commensal homeostasis which leads to improved overall survival. On a broader level, correction of dysbiosis with probiotic supplementation can serve as a preventive measure for war personnel at risk of exposure to radiation scenarios.

## Supplementary Information

**Additional file 1: Figure 1**. Mass calibration check for metabolomics experiment before and after running the samples (positive mode). Metmix shows a mass error window within 3 ppm.** Figure 2**. Mass calibration check for metabolomics experiment before and after running the samples (negative mode). Metmix shows a mass error window within 3 ppm.** Figure 3**. An overlay of all Total Ion Chromatogram (TIC) of all QC runs for metabolomics experiment (positive mode). Positive mode QC overlays showing minimal shifts in retention time and intensities.** Figure 4**. An overlay of all Total Ion Chromatogram (TIC) of all QC runs for metabolomics experiment (NEGATIVE mode). Negative mode QC overlays showing minimal shifts in retention time and intensities.** Figure 5**. Mass calibration check for lipidomic experiment before and after running the samples (positive mode). Metmix shows a mass error window within 5 ppm.** Figure 6**. Mass calibration check for lipidomic experiment before and after running the samples (negative mode). Metmix shows a mass error window within 5 ppm.** Figure 7**. An overlay of all Total Ion Chromatogram (TIC) of all QC runs for lipidomic experiment (positive mode). Positive mode QC overlays showing minimal shifts in retention time and intensities.** Figure 8**. An overlay of all Total Ion Chromatogram (TIC) of all QC runs for lipidomic experiment (negative mode). Negative mode QC overlays showing minimal shifts in retention time and intensities.** Table 1**. Results using the Adonis test comparing each group for all time points.** Table 2**. List of annotated metabolites/lipids with their corresponding chemical names, retention time, ionization mode, precursor mass, and CID fragments.** Table 3**. List of tandem MS validated metabolites from untargeted metabolomic analysis showing the test statistics for drug side effects, radiation effects, and BIO 300 protective effects.** Table 4**. The Mummichog (version 2.06) pathway analysis outcome for side effect.** Table 5**. The Mummichog pathway analysis outcome for radiation effect and BIO 300 protective effect at time SD 3.** Table 6**. The pathway analysis outcome for time SD 14 radiation effect and BIO 300 protective effect.

## Data Availability

All raw data in this study are openly available on the Dryad Digital Repository (https://doi.org/10.5061/dryad.hhmgqnkhb) and analyzed results are included in this published article (and its Supplementary Information Files).

## Abbreviations

ACN: Acetonitrile
API: Active pharmaceutical ingredient
ARS: Acute radiation syndrome
ASVs: Amplicon sequence variants
AUC: Area under the curve
Co: Cobalt
CID: Collision-induced dissociation
Cmax: Maximum plasma concentration
°C: Degree Celsius
DADA: Divisive Amplicon Denoising Algorithm
DNA: Deoxyribonucleic acid
EPR: Electron paramagnetic resonance
ERβ: Estrogen receptor-beta
ESI: Electrospray ionization
FC: Fold change
FDR: False discovery rate
γ: Gamma
g: Gravity
GI-ARS: Gastrointestinal acute radiation syndrome
Gy: Gray
h: Hour
H-ARS: Hematopoietic acute radiation syndrome
IACUC: Institutional Animal Care and Use Committee
IL-1β: Interleukin–1beta
IPO: Isotopologue parameter optimization
ITS: Internal transcribed spacer
kV: Kilovolts
L: Liter
LC–MS: Liquid chromatography–mass spectrometry
LD: Lethal Dose
µl: Microliter
mg: Milligram
ml: Milliliter
min: Minute
MS: Mass spectrometry
mz: Mass to charge
NIST: National Institute of Standards and Technology
NetCDF: Network Common Data Form
N: Normality
nm: Nanomolar
ng: Nanogram
OP: Oral powder
OS: Oral suspension
OTUs: Operational taxonomic unit
PCoA: Principal coordinate analysis
PCR: Polymerase chain reaction
pM: Picomolar
QC: Quality control
QToFQuadrupole-time-of-fl: Ight
rRNA: Ribosomal ribonucleic acid
ROS: Reactive oxygen species
rt: Retention time
SD: Study day
2D: Two-dimensional
TOF-MS: Time-of-flight mass spectrometry
Tris-Cl: Tris-Chloride
UCP: Ultra-Clean Production
UPLC: Ultra-performance liquid chromatography
v/v: Volume/volume
