# Supplementary material for: Microbiome study in irradiated mice treated with BIO 300, a promising radiation countermeasure
Source: Anim Microbiome. 2021 Oct 9;3:71. doi: 10.1186/s42523-021-00132-1 (PMC8501697; doi:10.1186/s42523-021-00132-1)
Supplement: Supplementary file 1 — Additional file 1: Figure 1. Mass calibration check for metabolomics experiment before and after running the samples (positive mode). Metmix shows a mass error window within 3 ppm. Figure 2. Mass calibration check for metabolomics experiment before and after running the samples (negative mode). Metmix shows a mass error window within 3 ppm. Figure 3. An overlay of all Total Ion Chromatogram (TIC) of all QC runs for metabolomics experiment (positive mode). Positive mode QC overlays showing minimal shifts in retention time and intensities. Figure 4. An overlay of all Total Ion Chromatogram (TIC) of all QC runs for metabolomics experiment (NEGATIVE mode). Negative mode QC overlays showing minimal shifts in retention time and intensities. Figure 5. Mass calibration check for lipidomic experiment before and after running the samples (positive mode). Metmix shows a mass error window within 5 ppm. Figure 6. Mass calibration check for lipidomic experiment before and after running the samples (negative mode). Metmix shows a mass error window within 5 ppm. Figure 7. An overlay of all Total Ion Chromatogram (TIC) of all QC runs for lipidomic experiment (positive mode). Positive mode QC overlays showing minimal shifts in retention time and intensities. Figure 8. An overlay of all Total Ion Chromatogram (TIC) of all QC runs for lipidomic experiment (negative mode). Negative mode QC overlays showing minimal shifts in retention time and intensities. Table 1. Results using the Adonis test comparing each group for all time points. Table 2. List of annotated metabolites/lipids with their corresponding chemical names, retention time, ionization mode, precursor mass, and CID fragments. Table 3. List of tandem MS validated metabolites from untargeted metabolomic analysis showing the test statistics for drug side effects, radiation effects, and BIO 300 protective effects. Table 4. The Mummichog (version 2.06) pathway analysis outcome for side effect. Table 5. The Mummichog pathway analys [file 42523_2021_132_MOESM1_ESM.pdf]

Initial			
Name	POS	INPUT	PPM error
Acetaminophen	152.0712	152.0710	-1.31
Sulfaguanidine	215.0603	215.0607	1.85
Sulfadimethoxine	311.0814	311.0815	0.32
Val-Tyr-Val	380.2185	380.2178	-1.84
Terfenadine	472.3216	472.3217	0.21
Leu-Enkephalin	556.2771	556.2775	0.71

Final			
Name	POS	INPUT	PPM error
Acetaminophen	152.0712	152.0712	0.00
Sulfaguanidine	215.0603	215.0604	0.46
Sulfadimethoxine	311.0814	311.0811	-0.96
Val-Tyr-Val	380.2185	380.2188	0.78
Terfenadine	472.3216	472.3228	2.54
Leu-Enkephalin	556.2771	556.2775	0.71

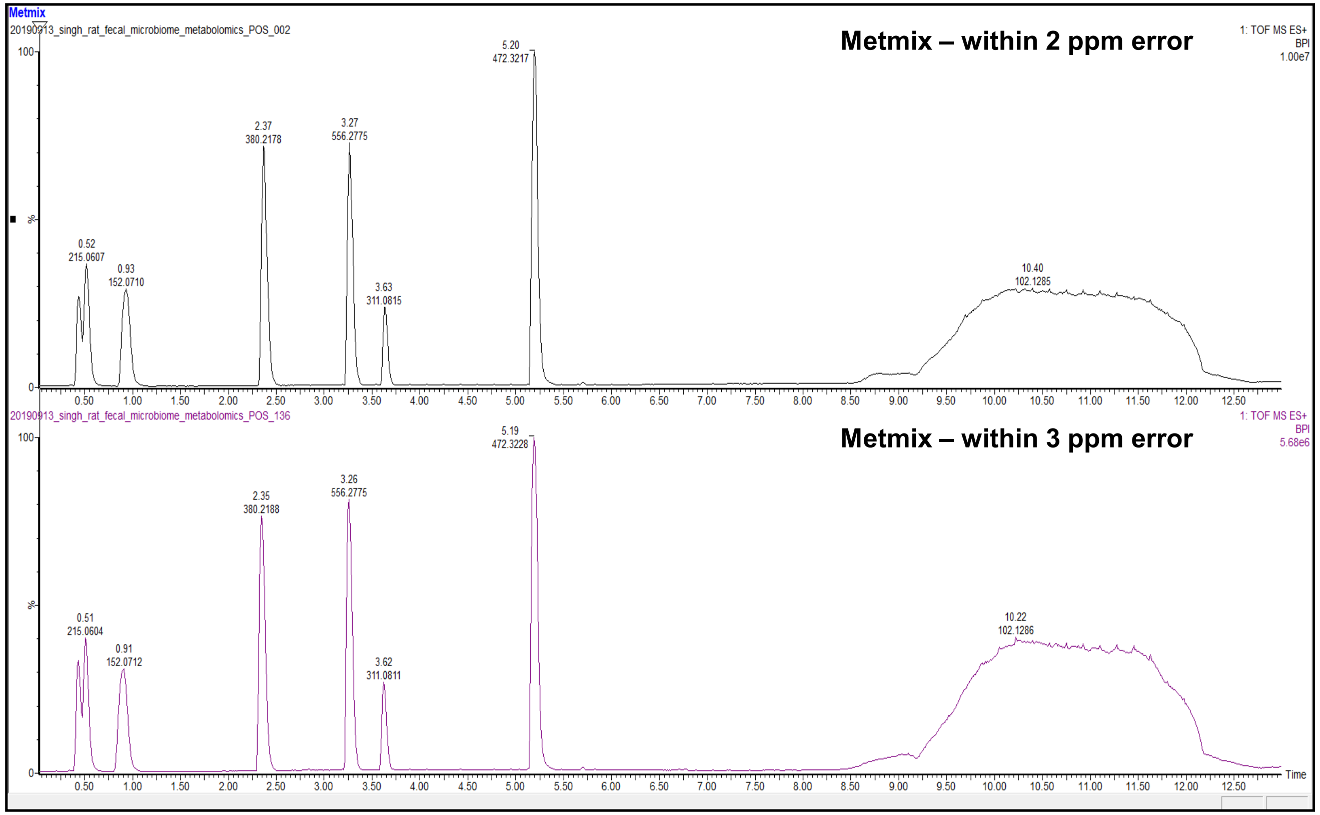

Additional file 1. Figure 1. Mass calibration check for metabolomics experiment before and after running the samples (positive mode). Metmix shows a mass error window within 3 ppm.

Initial			
Name	NEG	INPUT	PPM Error
Acetaminophen	150.0555	150.0551	-2.66
Sulfaguanidine	213.0446	213.0443	-1.40
Sulfadimethoxine	309.0658	309.0649	-2.91
Val-Tyr-Val	378.2029	378.2018	-2.90
Leu-Enkephalin	554.2615	554.2599	-2.88

Final			
Name	NEG	INPUT	PPM Error
Acetaminophen	150.0555	150.0556	0.66
Sulfaguanidine	213.0446	213.0450	1.87
Sulfadimethoxine	309.0658	309.0663	1.61
Val-Tyr-Val	378.2029	378.2023	-1.58
Leu-Enkephalin	554.2615	554.2612	-0.54

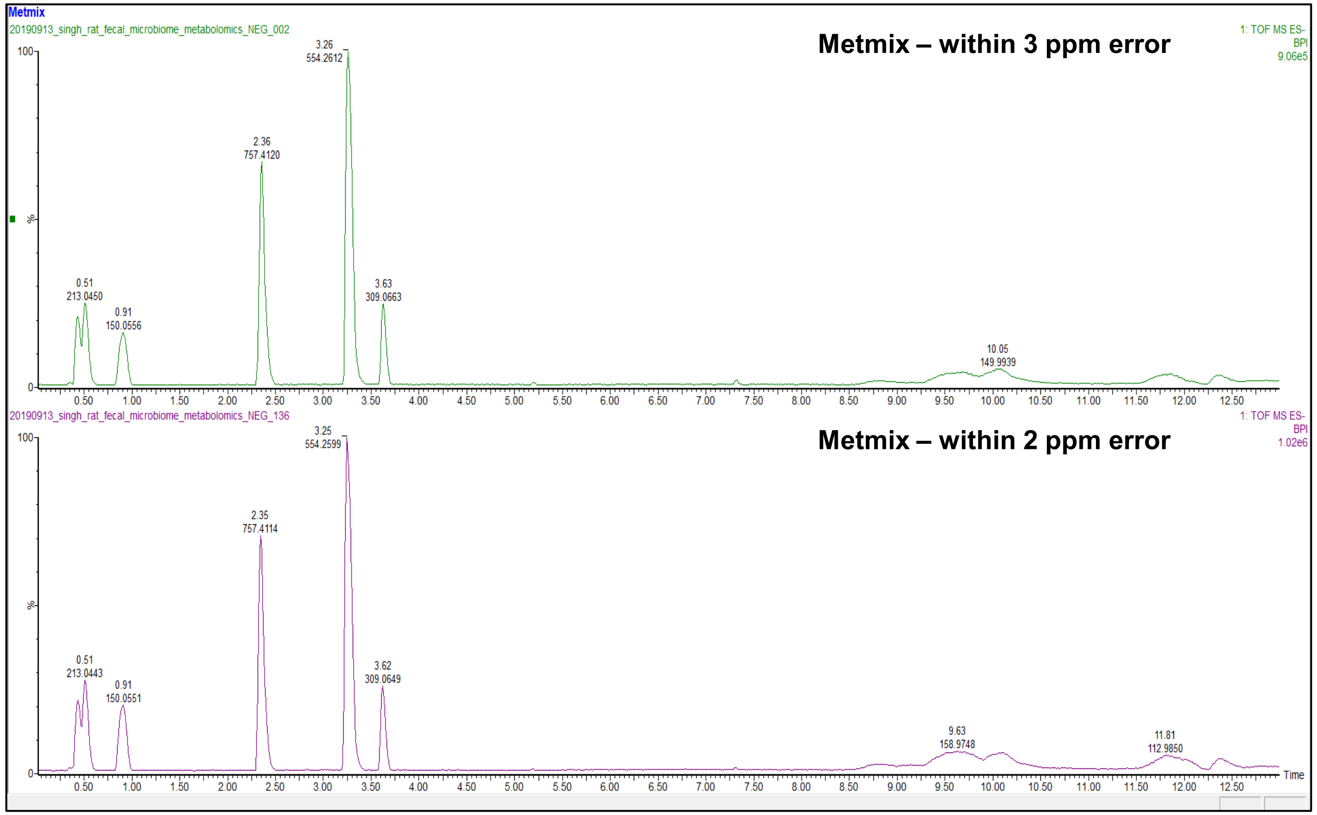

Additional file 1. Figure 2. Mass calibration check for metabolomics experiment before and after running the samples (negative mode). Metmix shows a mass error window within 3 ppm.

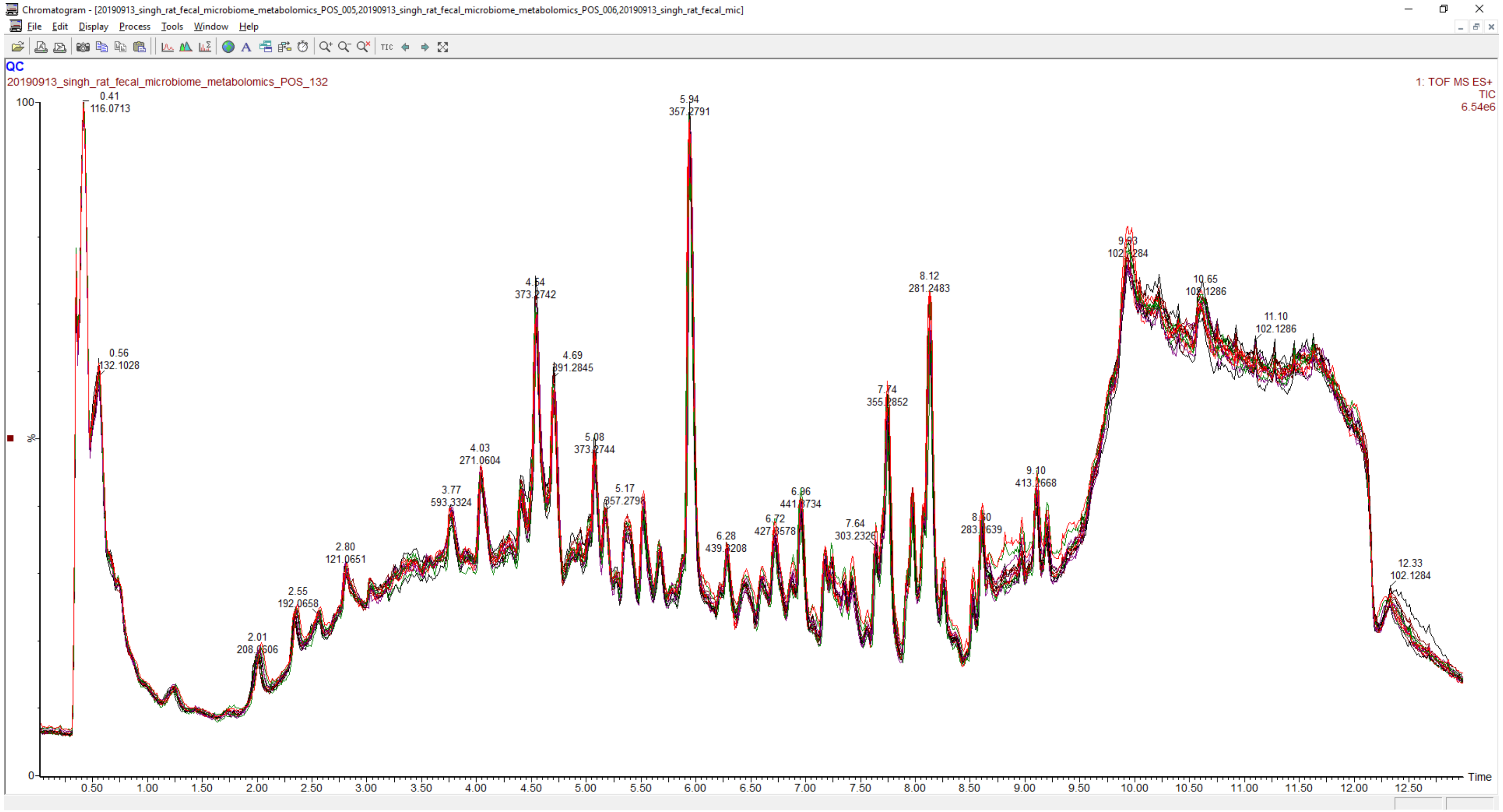

Additional file 1. Figure 3. An overlay of all Total Ion Chromatogram (TIC) of all QC runs for metabolomics experiment (positive mode). Positive mode QC overlays showing minimal shifts in retention time and intensities

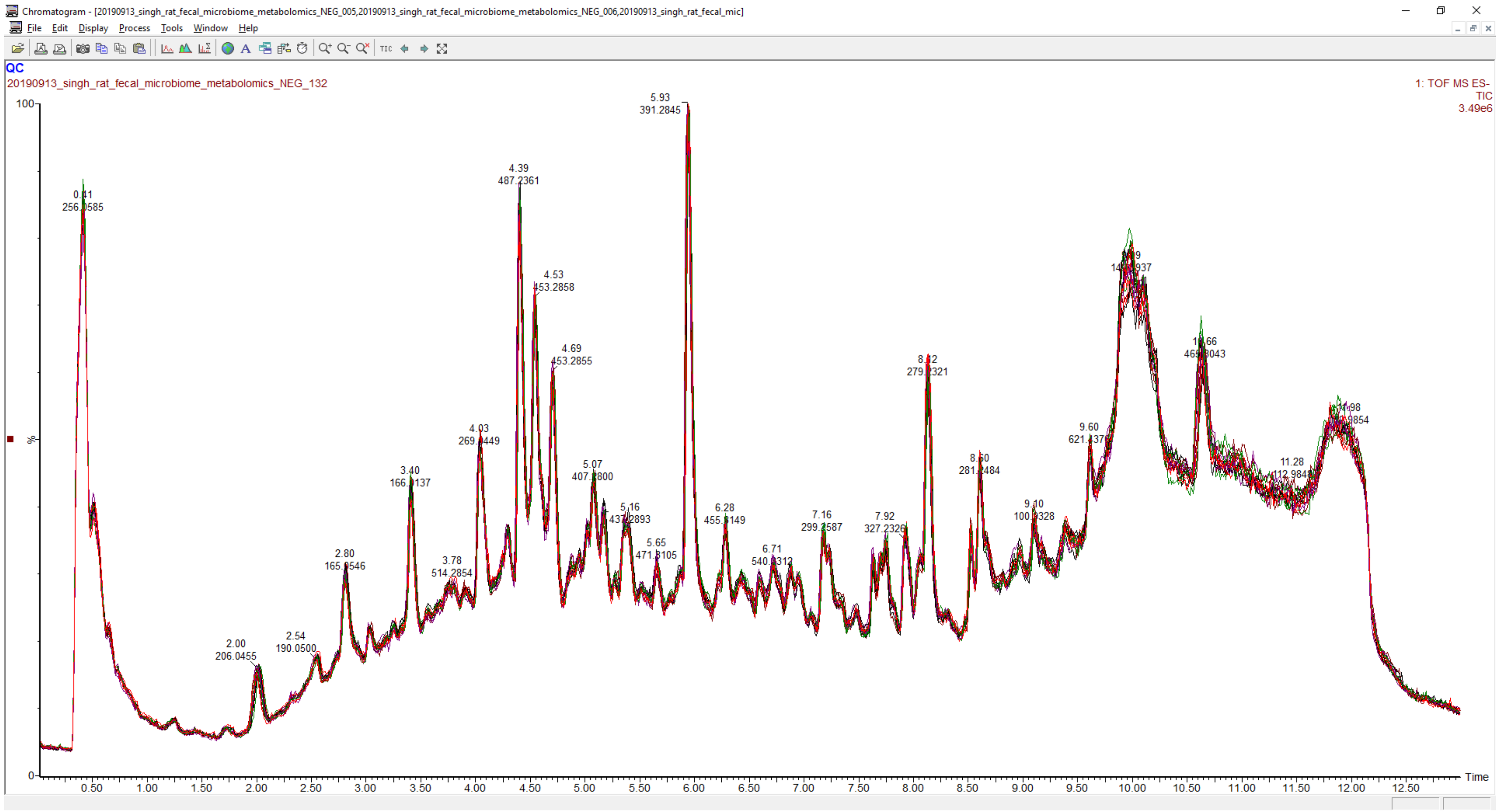

Additional file 1. Figure 4. An overlay of all Total Ion Chromatogram (TIC) of all QC runs for metabolomics experiment (NEGATIVE mode). Negative mode QC overlays showing minimal shifts in retention time and intensities

Initial			
Name	POS	INPUT	PPM error
Acetaminophen	152.0712	152.0713	0.65
Sulfaguanidine	215.0603	215.0604	0.46
Sulfadimethoxine	311.0814	311.0815	0.32
Val-Tyr-Val	380.2185	380.2187	0.52
Terfenadine	472.3216	472.3219	0.63
Leu-Enkephalin	556.2771	556.2770	-0.17

Final			
Name	POS	INPUT	PPM error
Acetaminophen	152.0712	152.0719	4.60
Sulfaguanidine	215.0603	215.0610	3.25
Sulfadimethoxine	311.0814	311.0822	2.57
Val-Tyr-Val	380.2185	380.2194	2.36
Terfenadine	472.3216	472.3224	1.69
Leu-Enkephalin	556.2771	556.2773	0.35

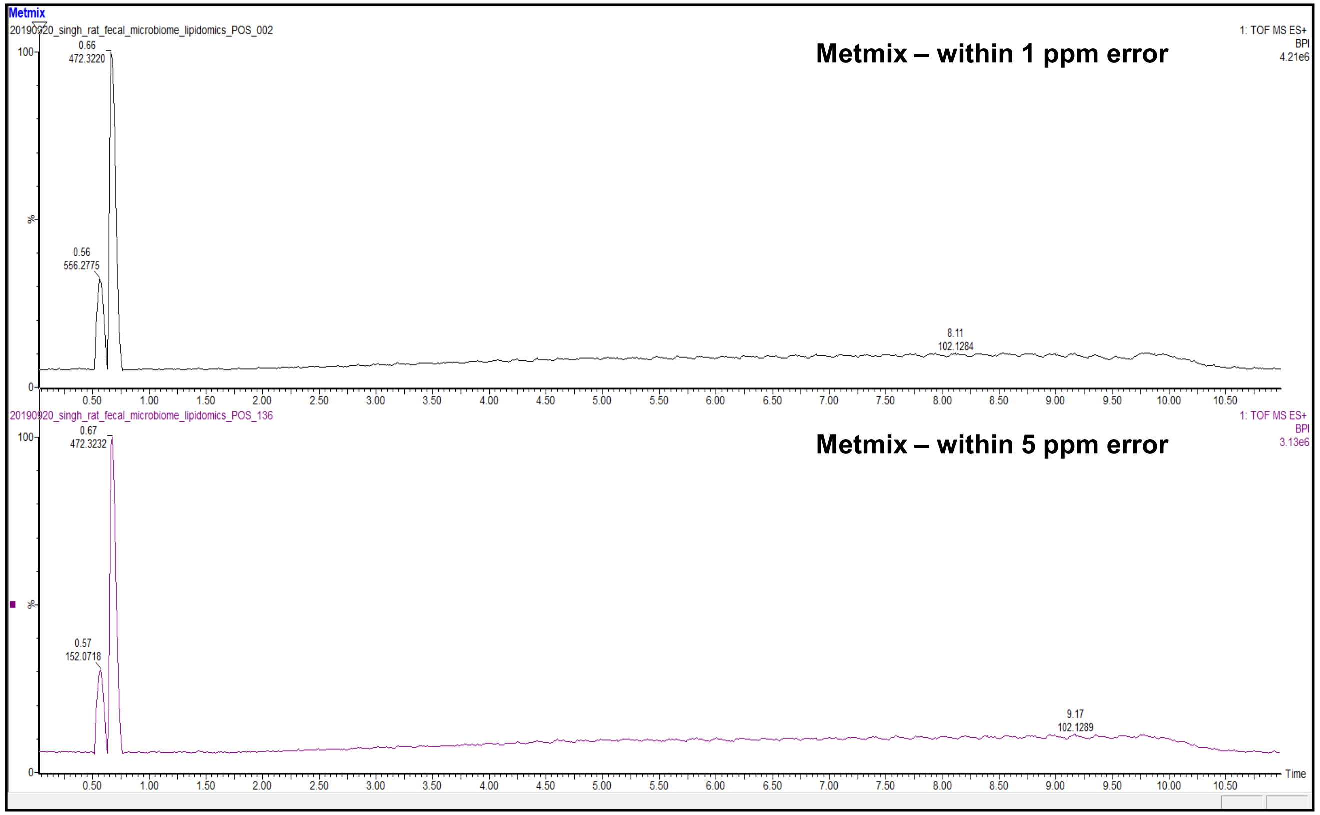

Additional file 1. Figure 5. Mass calibration check for lipidomic experiment before and after running the samples (positive mode). Metmix shows a mass error window within 5 ppm.

Initial			
Name	NEG	INPUT	PPM Error
Acetaminophen	150.0555	150.0562	4.66
Sulfaguanidine	213.0446	213.0454	3.75
Sulfadimethoxine	309.0658	309.0662	1.29
Val-Tyr-Val	378.2029	378.2038	2.37
Leu-Enkephalin	554.2615	554.2626	1.98

Final			
Name	NEG	INPUT	PPM Error
Acetaminophen	150.0555	150.0557	1.33
Sulfaguanidine	213.0446	213.0445	-0.46
Sulfadimethoxine	309.0658	309.0656	-0.64
Val-Tyr-Val	378.2029	378.2025	-1.05
Leu-Enkephalin	554.2615	554.2612	-0.54

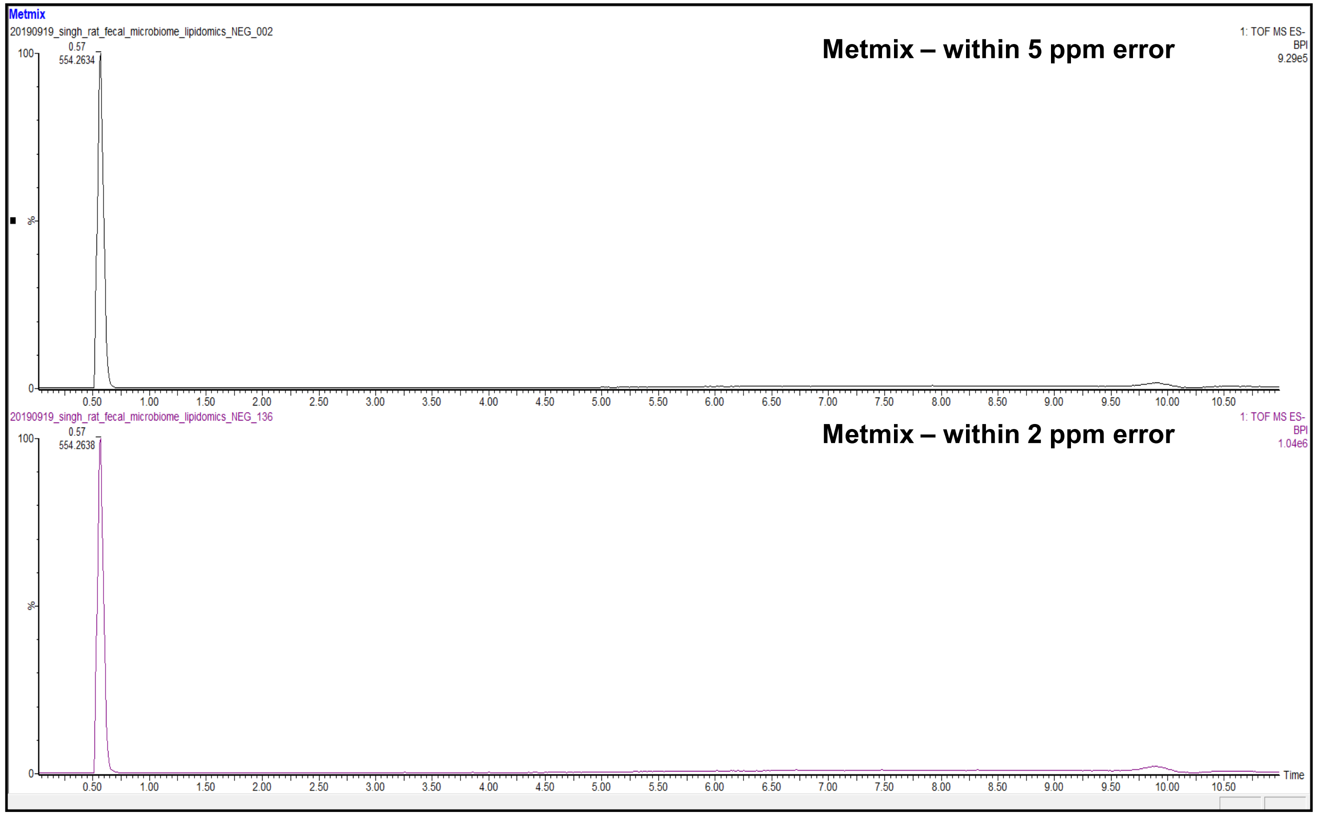

Additional file 1. Figure 6. Mass calibration check for lipidomic experiment before and after running the samples (negative mode). Metmix shows a mass error window within 5 ppm.

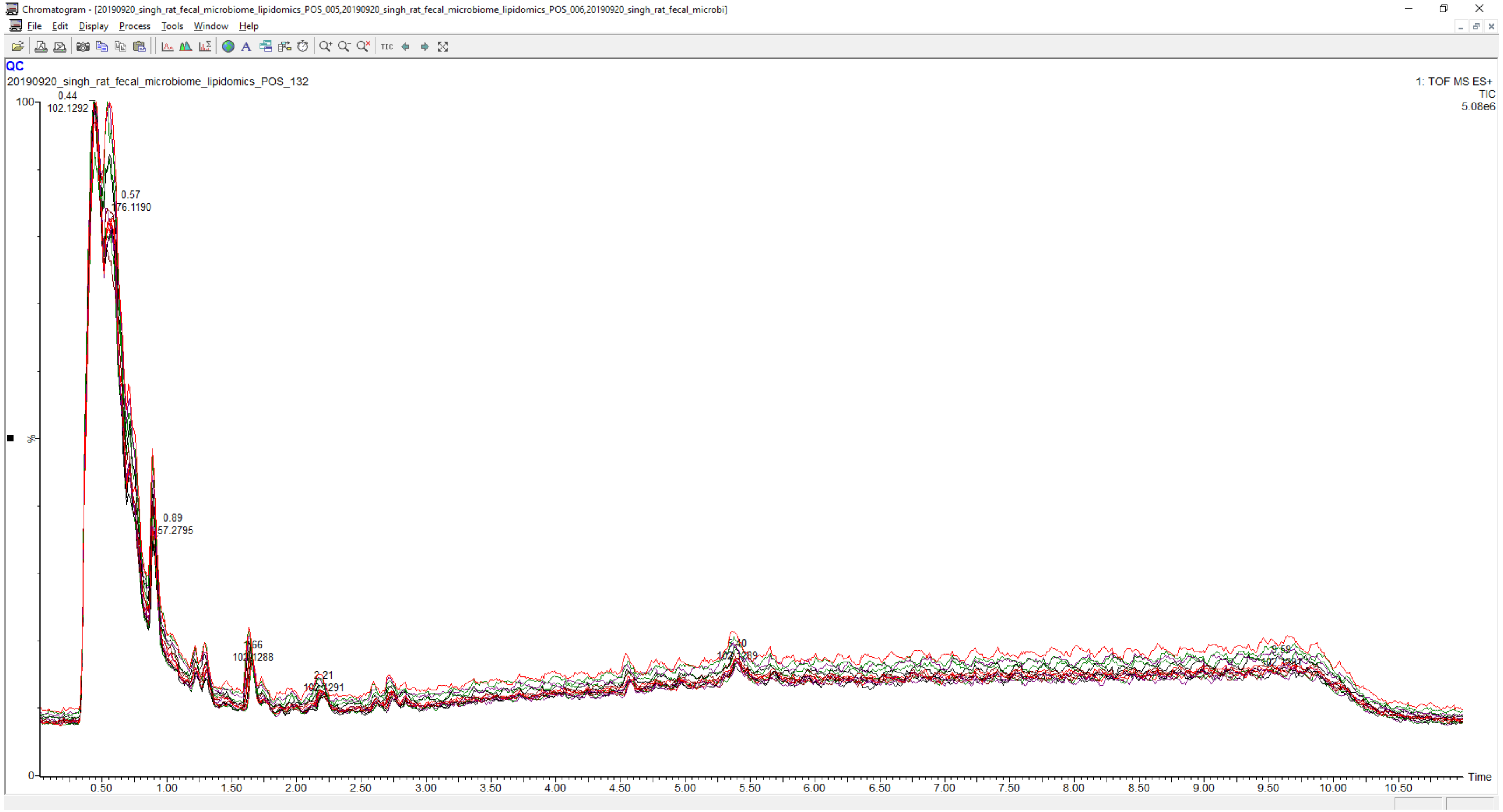

Additional file 1. Figure 7. An overlay of all Total Ion Chromatogram (TIC) of all QC runs for lipidomic experiment (positive mode). Positive mode QC overlays showing minimal shifts in retention time and intensities

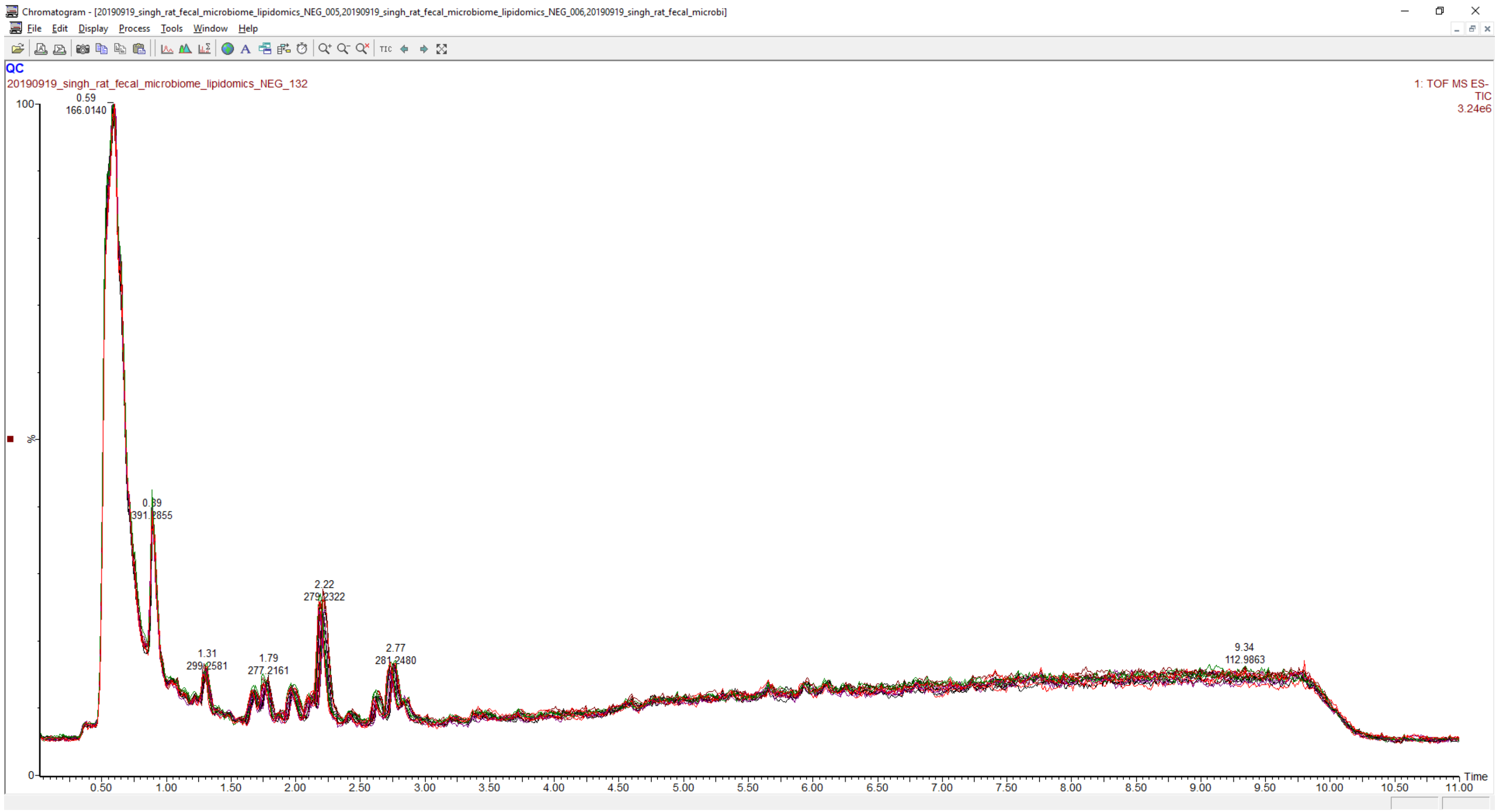

Additional file 1. Figure 8. An overlay of all Total Ion Chromatogram (TIC) of all QC runs for lipidomic experiment (negative mode). Negative mode QC overlays showing minimal shifts in retention time and intensities

Additional file 1. Table 1. Results using the Adonis test comparing each group for all time points

Group A	Group B	Day	Adonis p-value[1]
BIO 300 oral suspension	BIO 300 oral powder	-7	0.116
BIO 300 oral suspension	Control	-7	0.152
BIO 300 oral powder	Control	-7	0.513
BIO 300 oral suspension	BIO 300 oral powder	-1	**<0.001**
BIO 300 oral suspension	Control	-1	**<0.001**
BIO 300 oral powder	Control	-1	**<0.001**
BIO 300 oral suspension	BIO 300 oral powder	3	**0.016**
BIO 300 oral suspension	Control	3	**0.002**
BIO 300 oral powder	Control	3	**<0.001**
BIO 300 oral suspension	BIO 300 oral powder	14	**0.042**
BIO 300 oral suspension	Control	14	**0.007**
BIO 300 oral powder	Control	14	**<0.001**
BIO 300 oral suspension	BIO 300 oral powder	30	0.077
BIO 300 oral suspension	Control	30	NA[2]
BIO 300 oral powder	Control	30	NA[2]

[1] P-values less than 0.05 (bolded) were considered significant.
[2] Adonis test not applicable (Control n=2)

Additional file 1. Table 2. List of annotated metabolites/lipids with their corresponding chemical names, retention time, ionization mode, precursor mass, and CID fragments.

mode	precursor_mz_rt	m/z	RT	name	synonyms	adduct	level	cid	class
NEG	269.0448_4.04	269.0448	4.04	4',5,7-Trihydroxyisoflavone	Genistein	[M-H]-	2	269.05, 241.05, 240.04, 227.04, 225.06, 224.05, 213.06, 201.06, 199.04, 197.006, 181.07, 133.03, 107.01, 91.02, 63.02	
NEG	297.2426_7.36	297.2426	7.36	3-Oxooctadecanoic acid	3-Oxostearic acid	[M-H]-	2	297.24, 279.23, 265.0, 253.25, 183.01	
NEG	299.2582_7.18	299.2582	7.18	3-Hydroxyoctadecanoic acid	3-Hydroxystearic acid	[M-H]-	2	299.26, 253.25, 239.23	
NEG	391.2846_5.41	391.2846	5.41	Cholan-24-oic acid, 3,12-dihydroxy-, (3α,5β,12α	Deoxycholic acid	[M-H]-	2	391.28, 373.27, 355.26, 345.28, 343.26, 327.27, 317.28, 311.24, 69.03	Steroids and derivatives
NEG	389.2482_7.74	389.2482	7.74	12-Ketodeoxycholic acid	12-Oxolithocholic acid	[M-H]-	2	389.27, 371.26, 353.24, 345.28, 343.26	Steroids and derivatives
NEG	483.273_7.29	483.273	7.29	1-Palmitoyl-2-hydroxy-sn-glycero-3-phospho-	LysoPG(16:0)	[M-H]-	2	483.28, 255.23, 152.99, 96.96	
NEG	193.0498_2.87	193.0498	2.87	3-Hydroxy-4-methoxycinnamic acid	Ferulic acid	[M-H]-	2	193.05, 178.03, 149.06, 134.04	
NEG	480.3072_7.45	480.3072	7.45	1-Stearoyl-2-hydroxy-sn-glycero-3-phosphoetl	LysoPE(18:0)	[M-H]-	2	480.21, 283.26, 152.99	
NEG	509.2879_7.55	509.2879	7.55	1-Oleoyl-2-hydroxy-sn-glycero-3-phospho-(1'	PG(18:1/0:0)	[M-H]-	2	509.28, 281.25, 96.96	
NEG	165.0549_2.81	165.0549	2.81	3-(2-Hydroxyphenyl)propionic acid	Hydrocinnamic acid, o-hydroxy-	[M-H]-	2	165.055, 147.04, 121.06, 119.05, 106.04	
NEG	295.2271_6.49	295.2271	6.49	13-Hydroxy-9Z,11E-octadecadienoic acid	13-HODE	[M-H]-	2	295.22, 277.22, 251.23, 195.13, 171.10, 96.96	
NEG	293.2113_6.12	293.2113	6.12	9S-Hydroxy-10E,12Z,15Z-octadecatrienoic acid	9(S)-HOTrE	[M-H]-	2	293.21, 275.20, 195.13, 185.11, 171.10, 155.98, 96.96	
NEG	271.227_7.77	271.227	7.77	2-Hydroxypalmitic acid	Hexadecanoic acid, 2-hydroxy-	[M-H]-	2	271.22, 253.22, 225.22, 223.20, 73.00,	
NEG	253.2165_7.97	253.2165	7.97	16-Hydroxyhexadecanoic acid	16-Hydroxypalmitic acid	[M-H-H2O]-	2	253.21, 195.17, 132.03	
NEG	305.2478_8.5	305.2478	8.5	5α-Androstane-3,17-dione	DHA	[M+OH-]-	2	305.21, 174.95, 130.96, 86.97	Steroids and derivatives
NEG	178.0501_0.67	178.0501	0.67	Adrenochrome	1H-Indole-5,6-dione, 2,3-dihydro-3-hydroxy-1-methyl-	[M-H]-	3	178.05, 163.03, 134.02, 119.04, 108.02	
NEG	201.1236_0.97	201.1236	0.97	Ala-Ile	AI	[M-H]-	2	301.12, 164.83, 140.10, 130.08	
NEG	187.1077_0.99	187.1077	0.99	Alanylvaline	Ala-Val	[M-H]-	2	187.10, 143.11, 125.09, 116.07	
NEG	203.0819_1.22	203.0819	1.22	L-Tryptophan	Tryptophan	[M-H]-	2	203.08, 159.09, 142.06, 130.07, 116.05, 74.03	
NEG	253.0496_3.58	253.0496	3.58	7,3'-Dihydroxyflavone	4H-1-Benzopyran-4-one, 5-hydroxy-2-(3-hydroxyphenyl)-	[M-H]-	3	253.05, 225.06, 209.06, 135.00, 91.02	
NEG	164.071_0.73	164.071	0.73	L-Phenylalanine	Phe	[M-H]-	2	164.07, 147.04, 103.05, 72.00	
NEG	377.085_0.42	377.085	0.42	D-(+)-Lactose	Lactose	[M+Cl]-	2	377.08, 179.06, 161.04, 115.04, 101.02, 85.02	
NEG	389.269_5.94	389.269	5.94	12-Ketolithocholic acid	12-Ketodeoxycholic acid	[M-H]-	2	389.27, 387.25, 271.26, 353.25, 346.28, 345.28, 344.26, 343.26, 341.24, 325.25, 69.03	
NEG	131.0707_2.5	131.0707	2.5	2-Ethyl-2-hydroxybutyric acid	Butanoic acid, 2-ethyl-2-hydroxy-	[M-H]-	3	131.07, 85.06	
NEG	465.3038_10.65	465.3038	10.65	Cholesterol 3-sulfate	Cholesteryl sulfate	[M-H]-	3	465.31, 96.96	
NEG	452.2777_6.69	452.2777	6.69	1-Palmitoyl-2-hydroxy-sn-glycero-3-phosphoe	LysoPE(16:0)	[M-H]-	2	452.29, 255.23, 196.04, 153.00	
NEG	201.1125_3.76	201.1125	3.76	Sebacic acid	Decanedioic acid	[M-H]-	2	201.11, 183.10, 139.11	
NEG	492.2131_5.94	492.2131	5.94	1-(1,2R-Dihexadecanoylphosphothiotidyl)inosito	Ptd(S)Ins-(3,4)-P2 (1,2-dipalmitoyl)	[M-2H]2-	2	492.21, 255.23, 223.00, 158.92, 96.96	
NEG	514.2838_4.26	514.2838	4.26	Taurocholic acid	Ethanesulfonic acid, 2-[[(3α,5β,7α,12α)-3,7,12-trihydroxy-24-oxochol	[M-H]-	3	514.28, 124.00, 96.96	
NEG	830.5883_9.98	830.5883	9.98	1,2-dioleoyl-sn-glycero-3-phosphatidylcholine	1,2-Dioleoyl-sn-glycero-3-PC	[M+HCO2]-	3	830.59, 770.56, 281.24	
POS	323.256_7.19	323.256	7.19	1,11?,15S-Trihydroxyprost-13E-en-9-one	Prostaglandin E1 alcohol	[M+H-H2O]+	2	323.25, 305.21, 175.14, 135.11, 95.08, 81.06	Prostaglandines and derivatives
POS	321.2404_7.36	321.2404	7.36	11,12-Dihydroxy-5Z,8Z,14Z-eicosatrienoic acid	11,12-DHET	[M+H-H2O]+	2	321.24, 175.14, 149.13, 135.11, 121.1	Fatty acids and derivatives
POS	373.2352_6.94	373.2352	6.94	3α,7α,12α-Trihydroxy-5β-cholanic acid	3α,7α,12α-Trihydroxy-5β-cholanic acid	[M+H-2H2O]+	2	373.23, 213.1, 199.1	Steroids and derivatives
POS	405.2998_7.79	405.2998	7.79	Cholan-24-oic acid, 3,7,12-trihydroxy-, methyl e	Cholic acid methyl ester	[M+H-H2O]+	1	405.29, 369.27, 355.26, 337.25, 319.24, 226.96, 213.16, 147.11, 95.08	Steroids and derivatives
POS	391.2458_6.25	391.2458	6.25	Hyocholic acid	γ-Muricholic acid	[M+H-H2O]+	2	391.24, 355.26, 337.25, 319.24, 199.14, 159.11	Steroids and derivatives
POS	195.0044_11.59	195.0044	11.59	3-Hydroxy-4-methoxycinnamic acid	Isoferulic acid	[M+H]+	2	195, 177.05, 149.05, 145.02, 117.03, 89.03	Miscellaneous
POS	405.212_3.13	405.212	3.13	Cholan-24-oic acid, 3,7,12-trihydroxy-, methyl e	Cholic acid methyl ester	[M+H-H2O]+	2	405.21, 383.37, 355.36, 337.25, 319.29, 303.25, 227.15, 213.16, 189.14, 135.13, 159.11, 145.1, 119.08, 107.08	Steroids and derivatives
POS	357.2793_5.41	357.2793	5.41	β-Hyodeoxycholic acid	Isohyodeoxycholic acid	[M+H-2H2O]+	2	357.27, 339.26, 275.2, 347.16, 215.17, 195.13, 175.14, 161.13, 147.16,133.1, 119.08, 105.06	Steroids and derivatives
POS	137.1328_7.7	137.1328	7.7	2'-Deoxyinosine	Inosine, 2'-deoxy-	[M+H-C5H8O3]+	2	137.13, 19.03, 110.07	Miscellaneous
POS	305.2481_8.05	305.2481	8.05	cis-5,8,11,14-Eicosatetraenoic acid	Arachidonic acid	[M+H]+	1	305.24, 244.63, 159.11, 147.11, 133.1, 121.1, 107.06, 83.06	Fatty acids and derivatives
POS	391.2848_9.1	391.2848	9.1	Hyocholic acid	γ-Muricholic acid	[M+H-H2O]+	1	391.28, 355.26, 337.25, 319.24, 306.25, 189.14, 185.13, 175.15, 159.11, 145.1	Steroids and derivatives
POS	193.1224_2.73	193.1224	2.73	2-Dodecenedioic acid, (2E)-	trans-Traumatic acid	[M+H-2H2O]+	2	193.12, 175.11, 157.1, 147.16, 133.1, 117.06, 105.06, 91.06	Miscellaneous
POS	329.2318_4.29	329.2318	4.29	cis-4,7,10,13,16,19-Docosahexaenoic acid	Cervonic acid	[M+H]+	2	329.24, 311.23, 293.27, 283.2, 269.27, 255.17, 247.16, 237.16, 233.15, 215.17, 207.13, 201.16, 187.14, 175.14	Fatty acids and derivatives
POS	193.0693_2.57	193.0693	2.57	2-Dodecenedioic acid, (2E)-	trans-Traumatic acid	[M+H-2H2O]+	2	193.06, 1755.11, 165.12, 157.1, 147.11, 133.1, 119.05, 105.06	Miscellaneous
POS	369.228_4.29	369.228	4.29	Cholan-24-oic acid, 3,7,12-trihydroxy-, methyl e	Cholic acid methyl ester	[M+H-3H2O]+	2	369.22, 337.25, 319.24, 309.25, 273.18, 241.16, 227.17, 213.16, 189.14, 155.11, 145.1	Steroids and derivatives
POS	355.1553_6.09	355.1553	6.09	3α,7α,12α-Trihydroxy-5β-cholanic acid	Cholic acid	[M+H-3H2O]+	2	355.15, 159.11, 133.1, 81.06	Steroids and derivatives
POS	373.2116_7.93	373.2116	7.93	3α,7α,12α-Trihydroxy-5β-cholanic acid	Cholic acid	[M+H-2H2O]+	2	373.21, 254.1, 247.1, 239.1, 227.1, 213.1, 199.0	Steroids and derivatives
POS	373.2741_5.94	373.2741	5.94	3α,7α,12α-Trihydroxy-5β-cholanic acid	Cholic acid	[M+H-2H2O]+	2	373.21, 254.1, 247.1, 239.1, 227.1, 213.1, 199.0	Steroids and derivatives
POS	165.055_0.49	165.055	0.49	3-(4-Hydroxyphenyl)lactic acid	p-Hydroxyphenyllactic acid	[M+H-H2O]+	1	165.05, 147.04, 123.04, 119.04, 103.05, 95.05, 77.03	Miscellaneous
POS	353.2319_4.92	353.2319	4.92	9-Oxo-11α,15R,19R-trihydroxy-prost-13E-en-1	15(R),19(R)-HydroxypPG E1	[M+H-H2O]+	2	353.23, 299.2, 273.18, 215.14, 185.13, 159.11, 145.1, 135.11, 121.1, 107.08, 81.09	Miscellaneous
POS	301.2518_6.25	301.2518	6.25	14(15)-Epoxy-5Z,8Z,11Z,17Z-eicosatetraenoic ac	14(15)-EpETE	[M+H-H2O]+	2	301.25, 283.2, 257.13, 173.13, 161.13, 145.1, 133.1, 131.08, 121.1, 117.06, 105.06, 93.06, 81.06	Fatty acids and derivatives
POS	377.2676_1.65	377.2676	1.65	Prostaglandin E2 isopropyl ester	9-Oxo-11α,15S-dihydroxyprosta-5Z,13E-dien-1-oic acid, isopropyl e	[M+H-H2O]+	3	377.26, 359.25, 317. 253.19, 121.09, 95.09	Prostaglandins and Derivatives
POS	591.3182_0.63	591.3182	0.63	Urobilin	21H-Biline-8,12-dipropanoic acid, 3,18-diethyl-1,4,5,15,16,19,22,24-(	[M+H]+	2	591.32, 469.25, 468.25, 467.24, 466.23, 438.20, 394.21, 343.16, 285.15, 180.10	
POS	321.2412_1.31	321.2412	1.31	3β-Hydroxy-5-cholenoic acid	5-Cholenic acid-3β-ol	[M+H-3H2O]+	2	321.25, 279.21, 251.18, 225.16, 211.14, 185.13, 159.11, 95.08, 81.06	
POS	140.0712_0.57	140.0712	0.57	3-Amino-2,3-dihydrobenzoic acid	1,3-Cyclohexadiene-1-carboxylic acid, 5-amino-	[M+H]+	3	140.07, 122.06, 112.07, 94.06	
POS	337.2754_1.64	337.2754	1.64	8,11-Tridecadienoic acid, 13-(3-pentyl-2-oxiran	14(15)-EpEDE methyl ester	[M+H]+	2	337.27, 319.26, 269.22, 223.17, 173.13, 95.08, 81.07	Fatty Acids and Derivatives
POS	375.2521_1.32	375.2521	1.32	β-Hyodeoxycholic acid	Isohyodeoxycholic acid	[M+H-H2O]+	2	375.28, 357.27, 321.25, 275.19, 195.13, 161.13, 149.13, 121.10	
POS	522.3567_1.29	522.3567	1.29	1-Oleoyl-sn-glycero-3-phosphocholine	LysoPC(18:1)	[M+H]+	2	522.35, 504.34, 184.07, 104.11	
POS	376.2578_1.32	376.2578	1.32	N-Arachidonoyl-L-alanine	N-(1-Oxo-5Z,8Z,11Z,14Z-eicosatetraenyl)-L-alanine	[M+H]+	3	376.26, 302.18, 147.12, 133.01, 119.85, 105.07	
POS	546.3547_1.73	546.3547	1.73	1-Stearoyl-2-hydroxy-sn-glycero-3-phosphoch	LysoPC(18:0)	[M+Na]+	2	546.25, 487.27, 146.98, 104.10, 86.09	
POS	544.3387_1.29	544.3387	1.29	1-Oleoyl-sn-glycero-3-phosphocholine	LysoPC(18:1)	[M+Na]+	2	544.33, 485.26,146.98, 104.11, 86.09	
POS	303.2302_2.2	303.2302	2.2	Abietic acid	1- Phenanthrenecarboxylic acid, 1,2,3,4,4a,4b,5,6,7,9,10,10a-dodecahy	[M+H]+	1	303.23, 257.22, 201.16, 159.11, 121.1, 93.07, 81.07, 69.07	
POS	279.2331_0.97	279.2331	0.97	9(10)-Epoxy-12Z-octadecenoic acid	12(13)-EpOME	[M+H-H2O]+	2	279.23, 109.1, 95.08, 81.07, 67.05	
POS	277.2174_1.01	277.2174	1.01	13S-Hydroxy-9Z,11E,15Z-octadecatrienoic acid	13(S)-HOTrE	[M+H-H2O]+	2	277.21, 149.13, 135.11, 121.1, 107.08, 93.06, 81.07, 79.05, 67.05	
POS	295.228_1.01	295.228	1.01	17α-Hydroxyprogesterone	Pregn-4-ene-3,20-dione, 17-hydroxy-	[M+H-2H2O]+	2	295.20, 277.19, 253.19, 171.12, 145.10, 81.07	
POS	357.2798_0.91	357.2798	0.91	β-Hyodeoxycholic acid	Isohyodeoxycholic acid	[M+H-2H2O]+	1	357.27, 339.26, 321.25, 301.21, 275.20, 261.18, 247.16, 215.18, 161.13, 147.11, 135.11, 121.10, 109.10, 95.08, 81.07, 567.05	
NEG	351.2093_0.78	351.2093	0.78	15-Ketoprostaglandin F2α	9α,11α-Dihydroxy-15-oxoprosta-5Z,13E-dien-1-oic acid	[M-H]-	2	351.21, 333.21, 291.19, 279.19, 96.96, 79.95	
NEG	421.2346_0.81	421.2346	0.81	Dioctyl sulfosuccinate	Butanedionic acid, sulfo-, 1,4-bis(2-ethylhexyl) ester	[M-H]-	3	421.1, 227.2, 96.9, 80.9	
NEG	365.1867_0.64	365.1867	0.64	rac-2-Despiperidyl-2-aminorepaglinide	Benzoic acid, 4-[2-[[1-(2-aminophenyl)-3-methylbutyl]amino]-2-oxc	[M-H-H2O]-	2	365.19, 293.17, 291.15, 130.05, 96.96	

Additional file 1. Table 3: List of tandem MS validated metabolites from untargeted metabolomic analysis showing the test statistics for drug side effects, radiation effects, and BIO 300 protective effects.

			01. Bio300 OS Pre_7_vs_Vehicle_Pre_7				02. Bio300 OP Pre_7_vs_Vehicle_Pre_7			
**mz_RT**	**Name**	**Mode**	**p-value**	**FDR**	**FC**	**LOG2FC**	**p-value**	**FDR**	**FC**	**LOG2FC**
830.5883_9.98	1,2-Dioleoyl-sn-glycero-3-PC	NEG	0.99754	0.99973	1.1778	0.2362	0.92852	0.99839	1.0535	0.0751
140.0712_0.57	1,3-Cyclohexadiene-1-carboxylic acid, 5-amino-	POS	0.47327	0.99669	0.91067	-0.135	0.7342	0.99761	1.0385	0.0544
321.2404_7.36	11,12-DHET	POS	0.6331	0.99961	0.87002	-0.201	0.15781	0.99339	0.71737	-0.479
279.2331_0.97	12(13)-EpOME	POS	0.6999	0.99669	1.1077	0.1476	0.14758	0.99761	1.2538	0.3263
389.269_5.94	12-Ketodeoxycholic acid	NEG	0.34838	0.99973	0.77554	-0.367	0.008807	0.57167	0.50565	-0.984
389.2482_7.74	12-Oxolithocholic acid	NEG	0.8848	0.99973	1.0968	0.1333	0.35492	0.99839	1.1796	0.2384
277.2174_1.01	13(S)-HOTrE	POS	0.9351	0.99669	1.0751	0.1045	0.09479	0.99761	1.392	0.4771
295.2271_6.49	13-HODE	NEG	0.95505	0.99973	1.0163	0.0233	0.73724	0.99839	1.0914	0.1262
337.2754_1.64	14(15)-EpEDE methyl ester	POS	0.74645	0.99669	0.92085	-0.119	0.5281	0.99761	1.1708	0.2275
301.2518_6.25	14(15)-EpETE	POS	0.79652	0.99961	1.4511	0.5371	0.53134	0.99767	0.76645	-0.384
353.2319_4.92	15(R),19(R)-HydroxypPG E1	POS	0.93659	0.99961	0.98112	-0.028	0.26532	0.99767	0.78405	-0.351
253.2165_7.97	16-Hydroxypalmitic acid	NEG	0.60425	0.99973	0.94653	-0.079	0.35852	0.99839	0.86704	-0.206
271.227_7.77	2-(OH)-Hexadecanoic acid	NEG	0.35616	0.99973	0.66757	-0.583	0.86138	0.99839	1.1052	0.1443
137.1328_7.7	2-(OH)-Inosine	POS	0.86704	0.99961	0.97871	-0.031	0.9941	0.99909	0.96233	-0.055
178.0501_0.67	3(OH)-1-Me-2,3-dihydroindole-5,6-dione	NEG	0.46951	0.99973	1.6253	0.7007	0.22241	0.99839	0.77069	-0.376
299.2582_7.18	3-(OH)-stearic acid	NEG	0.33244	0.99973	0.82868	-0.271	0.037503	0.80672	0.6016	-0.733
297.2426_7.36	3-Oxostearic acid	NEG	0.55206	0.99973	0.84345	-0.246	0.13232	0.99839	0.67816	-0.56
373.2352_6.94	3α,7α,12α-(OH)3-5β-cholanic acid	POS	0.93242	0.99961	0.95949	-0.06	0.37872	0.99767	1.1059	0.1452
321.2412_1.31	5-Cholenic acid-3β-ol	POS	0.42932	0.99669	0.7929	-0.335	0.079047	0.97494	0.60628	-0.722
293.2113_6.12	9(S)-HOTrE	NEG	0.85811	0.99973	1.0626	0.0877	0.098191	0.99839	1.2938	0.3716
351.2093_0.78	9α,11α-Dihydroxy-15-oxoprosta-5Z,13E-dien-1-oic acid	NEG	0.43863	0.99009	1.2711	0.346	0.95238	0.99886	0.90242	-0.148
201.1236_0.97	Al	NEG	0.83524	0.99973	1.0246	0.035	0.29245	0.99839	0.7415	-0.431
187.1077_0.99	Ala-Val	NEG	0.89877	0.99973	0.97186	-0.041	0.64822	0.99839	0.81142	-0.301
305.2481_8.05	Arachidonic acid	POS	0.73424	0.99961	1.0032	0.0047	0.26791	0.99767	0.76431	-0.388
365.1867_0.64	Benzoic acid, 4-[2-[[1-(2-aminophenyl)-3-methylbutyl]amino]-2-oxoethyl]-2-ethoxy-	NEG	0.2214	0.99009	1.2299	0.2986	0.96675	0.99886	0.99156	-0.012
421.2346_0.81	Butanedionic acid, sulfo-, 1,4-bis(2-ethylhexyl) ester	NEG	0.53464	0.99009	0.93039	-0.104	0.94645	0.99886	1.0014	0.002
131.0707_2.5	Butanoic acid, 2-ethyl-2-hydroxy-	NEG	0.13345	0.99973	0.4123	-1.278	0.03225	0.80672	2.4866	1.3142
329.2318_4.29	Cervonic acid	POS	0.31403	0.99961	0.8198	-0.287	0.060291	0.98077	0.67447	-0.568
465.3038_10.65	Cholesteryl sulfate	NEG	0.81821	0.99973	1.3647	0.4486	0.49042	0.99839	1.4778	0.5634
373.2116_7.93	Cholic acid	POS	0.73247	0.99961	1.093	0.1283	0.64911	0.99767	0.92629	-0.11
369.228_4.29	Cholic acid methyl ester	POS	0.45379	0.99961	0.8492	-0.236	0.13786	0.99339	0.73018	-0.454
201.1125_3.76	Decanedioic acid	NEG	0.73811	0.99973	0.91802	-0.123	0.30295	0.99839	0.81069	-0.303
391.2846_5.41	Deoxycholic acid	NEG	0.6419	0.99973	1.1387	0.1873	0.97421	0.99839	1.0207	0.0296
305.2478_8.5	DHA	NEG	0.12678	0.99973	0.68258	-0.551	0.22328	0.99839	0.719	-0.476
514.2838_4.26	Ethanesulfonic acid, 2-[[(3α,5β,7α,12α)-3,7,12-trihydroxy-24-oxocholan-24-yl]amino]-	NEG	0.62566	0.99973	0.9194	-0.121	0.88961	0.99839	0.86776	-0.205
193.0498_2.87	Ferulic acid	NEG	0.72768	0.99973	0.82525	-0.277	0.41456	0.99839	1.3697	0.4539
269.0448_4.04	Genistein	NEG	0.48182	0.99973	0.96563	-0.05	0.5696	0.99839	0.93228	-0.101
195.0044_11.59	Isoferulic acid	POS	0.28782	0.99961	0.79369	-0.333	0.46907	0.99767	0.84079	-0.25
375.2521_1.32	Isohyodeoxycholic acid	POS	0.63005	0.99669	0.76692	-0.383	0.65711	0.99761	1.0099	0.0142
253.0496_3.58	Jaranol	NEG	0.4193	0.99973	1.2142	0.28	0.52919	0.99839	1.0895	0.1236
377.085_0.42	Lactose	NEG	0.37732	0.99973	1.23	0.2986	0.48528	0.99839	1.3504	0.4334
546.3547_1.73	LysoPC(18:0)	POS	0.89783	0.99669	1.2584	0.3316	0.97504	0.99761	0.97472	-0.037
522.3567_1.29	LysoPC(18:1)	POS	0.53357	0.99669	0.93848	-0.092	0.67002	0.99761	1.0522	0.0734
452.2777_6.69	LysoPE(16:0)	NEG	0.6298	0.99973	1.1916	0.2529	0.57213	0.99839	0.79983	-0.322
480.3072_7.45	LysoPE(18:0)	NEG	0.68718	0.99973	1.4403	0.5264	0.98635	0.99839	0.94519	-0.081
483.273_7.29	LysoPG(16:0)	NEG	0.63743	0.99973	0.9661	-0.05	0.034369	0.80672	0.56697	-0.819
376.2578_1.32	N-(1-Oxo-5Z,8Z,11Z,14Z-eicosatetraenyl)-L-alanine	POS	0.40313	0.99669	0.76748	-0.382	0.47764	0.99761	1.0964	0.1328
303.2302_2.2	Neoabietic acid	POS	0.83911	0.99669	0.96398	-0.053	0.78803	0.99761	0.89671	-0.157
165.0549_2.81	o-hydroxy-Hydrocinnamic acid	NEG	0.69103	0.99973	0.96262	-0.055	0.72584	0.99839	0.92439	-0.113
509.2879_7.55	PG(18:1/0:0)	NEG	0.65954	0.99973	0.97583	-0.035	0.13766	0.99839	0.54091	-0.887
323.256_7.19	PGE1 alcohol	POS	0.48768	0.99961	0.85616	-0.224	0.13185	0.99332	0.71178	-0.491
377.2676_1.65	PGE2 Isopropyl ester	POS	0.96366	0.99669	0.84133	-0.249	0.33308	0.99761	1.1387	0.1874
164.071_0.73	Phe	NEG	0.72386	0.99973	1.0203	0.029	0.95478	0.99839	1.032	0.0454
165.055_0.49	p-Hydroxyphenyllactic acid	POS	0.62567	0.99961	1.0921	0.1271	0.93943	0.99767	1.0836	0.1158
295.228_1.01	Pregn-4-ene-3,20-dione, 17-hydroxy-	POS	0.89018	0.99669	1.1188	0.162	0.094624	0.99761	1.4281	0.5141
492.2131_5.94	Ptd(S)Ins-(3,4)-P2 (1,2-dipalmitoyl)	NEG	0.64374	0.99973	1.577	0.6572	0.80509	0.99839	1.1538	0.2064
193.0693_2.57	trans-Traumatic acid	POS	0.84313	0.99961	0.9856	-0.021	0.46488	0.99767	1.0726	0.1012
203.0819_1.22	Tryptophan	NEG	0.34974	0.99973	1.2504	0.3224	0.73056	0.99839	1.0471	0.0663
591.3182_0.63	Urobilin	POS	0.095718	0.99669	0.47989	-1.059	0.72526	0.99761	0.7479	-0.419
391.2848_9.1	γ-Muricholic acid	POS	0.37379	0.99961	1.0995	0.1368	0.61195	0.99767	1.0319	0.0453

	03. Bio300 OS Pre_1_vs_Vehicle_Pre_1				04. Bio300 OP Pre_1_vs_Vehicle_Pre_1				05. Vehicle_SD_3_vs_Vehicle_Pre_1			
**mz_RT**	**p-value**	**FDR**	**FC**	**LOG2FC**	**p-value**	**FDR**	**FC**	**LOG2FC**	**p-value**	**FDR**	**FC**	**LOG2FC**
830.5883_9.98	0.33758	0.64791	0.64953	-0.623	0.22498	0.65378	0.54405	-0.878	0.029191	0.07269	0.3411	-1.552
140.0712_0.57	0.051665	0.30722	0.74796	-0.419	0.014495	0.58809	0.64479	-0.633	9.7E-06	0.000315	0.46767	-1.096
321.2404_7.36	0.012988	0.1322	1.8161	0.8608	0.37828	0.72892	1.3036	0.3825	2.86E-05	0.000952	4.1659	2.0586
279.2331_0.97	0.1308	0.40784	0.73086	-0.452	0.33971	0.68853	1.2201	0.287	0.95785	0.977	1.013	0.0186
389.269_5.94	0.71859	0.87794	1.0201	0.0287	0.001147	0.14551	0.64893	-0.624	0.25176	0.36824	0.86769	-0.205
389.2482_7.74	0.10377	0.38849	0.70308	-0.508	0.18271	0.62084	0.74696	-0.421	0.000787	0.006958	0.41929	-1.254
277.2174_1.01	0.014886	0.17951	0.5517	-0.858	0.91527	0.96387	0.99524	-0.007	0.61266	0.70441	0.89528	-0.16
295.2271_6.49	0.23839	0.55229	0.79317	-0.334	0.24298	0.66107	1.1632	0.2181	0.94146	0.96061	1.0264	0.0376
337.2754_1.64	0.088721	0.34709	0.62676	-0.674	0.1111	0.61033	0.62246	-0.684	1.64E-05	0.000433	0.19117	-2.387
301.2518_6.25	0.12725	0.33415	0.65488	-0.611	0.98734	0.9971	0.94791	-0.077	2.61E-06	0.000265	10.366	3.3738
353.2319_4.92	0.79402	0.88326	1.0301	0.0427	0.75646	0.90875	1.0849	0.1176	0.26239	0.36148	0.9936	-0.009
253.2165_7.97	0.14652	0.43393	0.84525	-0.243	0.54009	0.79171	0.92564	-0.111	0.018975	0.052452	0.6963	-0.522
271.227_7.77	0.54801	0.78184	0.93835	-0.092	0.033723	0.41027	0.56546	-0.822	0.73566	0.80518	2.0352	1.0252
137.1328_7.7	0.022442	0.1607	0.75228	-0.411	0.91663	0.96945	0.97458	-0.037	0.02048	0.050416	0.70657	-0.501
178.0501_0.67	0.21009	0.5243	0.7977	-0.326	0.47133	0.76063	1.261	0.3346	0.052023	0.11216	0.66844	-0.581
299.2582_7.18	0.063574	0.31293	1.6219	0.6977	0.38127	0.72307	1.1649	0.2203	0.000116	0.002145	3.5596	1.8317
297.2426_7.36	0.012076	0.1826	1.8232	0.8665	0.33682	0.70002	1.3502	0.4331	9.77E-05	0.001915	4.4643	2.1584
373.2352_6.94	0.13904	0.34778	0.73457	-0.445	0.45215	0.7633	0.89679	-0.157	8.24E-05	0.001522	0.39878	-1.326
321.2412_1.31	0.018347	0.18985	1.9647	0.9743	0.69207	0.89263	1.2001	0.2631	6.86E-06	0.000245	5.8184	2.5406
293.2113_6.12	0.15308	0.4448	0.73818	-0.438	0.46476	0.75875	1.0905	0.125	0.43068	0.55075	0.85551	-0.225
351.2093_0.78	0.003872	0.30015	1.5572	0.6389	0.51643	0.95186	1.4093	0.495	1.53E-06	0.000291	2.5188	1.3327
201.1236_0.97	0.18542	0.49184	1.402	0.4875	0.53818	0.79171	1.082	0.1138	0.04298	0.098476	0.63917	-0.646
187.1077_0.99	0.19724	0.50386	1.9523	0.9652	0.39189	0.72868	1.0894	0.1235	0.052327	0.11248	0.79757	-0.326
305.2481_8.05	0.00526	0.10377	0.73613	-0.442	0.037358	0.47521	0.80447	-0.314	0.053812	0.10418	0.82469	-0.278
365.1867_0.64	0.023078	0.39275	1.4961	0.5812	0.067661	0.82429	1.3606	0.4443	0.003698	0.049537	1.6848	0.7526
421.2346_0.81	0.42581	0.80989	1.0665	0.0929	0.85514	0.98293	1.0175	0.025	6.97E-06	0.000828	1.766	0.8205
131.0707_2.5	0.68064	0.86132	0.76524	-0.386	0.70313	0.86479	1.1993	0.2622	0.16187	0.26825	0.8562	-0.224
329.2318_4.29	0.11096	0.31641	0.70758	-0.499	0.53086	0.802	0.87723	-0.189	0.06748	0.12413	0.66306	-0.593
465.3038_10.65	0.4278	0.70841	0.77544	-0.367	0.14375	0.58025	0.52778	-0.922	0.20436	0.31758	0.65213	-0.617
373.2116_7.93	0.020731	0.15464	0.66634	-0.586	0.64447	0.85732	0.92361	-0.115	0.26232	0.36148	0.81961	-0.287
369.228_4.29	0.36151	0.56731	0.87918	-0.186	0.67071	0.86919	0.96861	-0.046	0.2718	0.37233	0.84047	-0.251
201.1125_3.76	0.27778	0.58912	1.1297	0.176	0.49865	0.77178	0.90936	-0.137	0.11143	0.20053	0.73041	-0.453
391.2846_5.41	0.88103	0.94435	1.0228	0.0325	0.60401	0.81442	1.1242	0.1689	0.004564	0.019798	1.8525	0.8895
305.2478_8.5	0.45265	0.72601	0.85305	-0.229	0.77761	0.90184	0.99287	-0.01	0.24971	0.366	1.9976	0.9982
514.2838_4.26	0.077197	0.34388	0.64759	-0.627	0.45537	0.75611	0.8654	-0.209	0.81327	0.86502	5.278	2.4
193.0498_2.87	0.39372	0.68402	0.69958	-0.515	0.58912	0.80856	1.0434	0.0613	0.003764	0.017794	0.25449	-1.974
269.0448_4.04	0.000197	0.047874	126.85	6.987	0.000284	0.10262	17.474	4.1271	0.84488	0.89074	1.3783	0.4629
195.0044_11.59	0.020286	0.1536	1.5335	0.6168	0.10658	0.57051	1.5289	0.6125	0.003232	0.014508	1.6369	0.7109
375.2521_1.32	0.039056	0.26964	0.60634	-0.722	0.17334	0.61033	0.75798	-0.4	4.18E-05	0.000852	0.26455	-1.918
253.0496_3.58	0.79585	0.90738	1.1741	0.2316	0.82589	0.92407	0.8006	-0.321	0.25623	0.37326	1.0159	0.0227
377.085_0.42	0.71641	0.87794	1.4122	0.498	0.53283	0.79009	0.98318	-0.024	0.1168	0.20828	0.56049	-0.835
546.3547_1.73	0.11298	0.37694	0.36793	-1.443	0.14359	0.61033	0.26761	-1.902	0.000529	0.0045	0.046831	-4.416
522.3567_1.29	0.17474	0.44087	0.65761	-0.605	0.17411	0.61033	0.5942	-0.751	6.36E-05	0.001089	0.16245	-2.622
452.2777_6.69	0.51277	0.76404	0.93135	-0.103	0.89391	0.95737	1.0074	0.0106	0.1037	0.19011	0.73799	-0.438
480.3072_7.45	0.68619	0.86365	1.119	0.1622	0.37846	0.71992	0.62234	-0.684	0.015447	0.045479	0.28999	-1.786
483.273_7.29	0.77791	0.89818	0.99823	-0.003	0.091708	0.51233	0.75934	-0.397	0.001148	0.008775	0.51555	-0.956
376.2578_1.32	0.049051	0.30464	0.68928	-0.537	0.19493	0.61033	0.81883	-0.288	8.2E-05	0.001273	0.38278	-1.385
303.2302_2.2	0.023262	0.22125	0.78126	-0.356	0.33973	0.68853	0.92475	-0.113	0.002373	0.013135	0.58821	-0.766
165.0549_2.81	0.013974	0.18952	2.5166	1.3315	0.004982	0.24019	1.8421	0.8814	0.016631	0.04813	0.46727	-1.098
509.2879_7.55	0.80858	0.91272	1.0743	0.1033	0.16029	0.59228	0.77456	-0.369	0.015797	0.046087	0.61237	-0.708
323.256_7.19	0.18013	0.39725	1.5045	0.5893	0.36529	0.7221	1.1699	0.2264	2.24E-05	0.000845	3.6693	1.8755
377.2676_1.65	0.051901	0.30722	0.59514	-0.749	0.11936	0.61033	0.67005	-0.578	6.8E-07	6.86E-05	0.16432	-2.605
164.071_0.73	0.18299	0.48898	1.5898	0.6688	0.58109	0.80568	1.0207	0.0296	0.042308	0.097451	0.60317	-0.729
165.055_0.49	0.25049	0.46957	1.506	0.5907	0.38403	0.73051	1.0983	0.1353	0.19287	0.28435	0.82108	-0.284
295.228_1.01	0.023088	0.22125	0.59382	-0.752	0.57038	0.85199	1.1921	0.2535	0.79882	0.86418	0.96694	-0.049
492.2131_5.94	0.05507	0.29343	0.59881	-0.74	0.9597	0.98272	1.3701	0.4543	0.060052	0.12445	2.1548	1.1076
193.0693_2.57	0.10309	0.30805	0.76451	-0.387	0.065892	0.56368	0.72473	-0.464	0.10494	0.17282	0.77592	-0.366
203.0819_1.22	0.70898	0.87717	1.0836	0.1159	0.68648	0.8594	0.84221	-0.248	0.0516	0.1117	0.56626	-0.82
591.3182_0.63	0.18418	0.45099	1.4911	0.5764	0.48817	0.79542	1.0752	0.1046	4.8E-06	0.000201	16.215	4.0192
391.2848_9.1	0.85854	0.91705	1.0037	0.0053	0.8441	0.94965	1.0149	0.0214	0.097788	0.1643	1.0735	0.1023

	06. Bio300 OS SD_3_vs_Vehicle_SD_3				07. Bio300 OP SD_3_vs_Vehicle_SD_3			
**mz_RT**	**p-value**	**FDR**	**FC**	**LOG2FC**	**p-value**	**FDR**	**FC**	**LOG2FC**
830.5883_9.98	0.85969	0.93234	0.87944	-0.185	0.072358	0.18815	1.7706	0.8242
140.0712_0.57	0.90563	0.95107	1.0796	0.1105	0.21367	0.43097	0.88132	-0.182
321.2404_7.36	0.032252	0.093322	2.5505	1.3508	0.018575	0.086937	2.2716	1.1837
279.2331_0.97	6.71E-05	0.005019	0.23811	-2.07	0.022237	0.13122	0.5124	-0.965
389.269_5.94	0.15328	0.29558	0.7354	-0.443	0.43658	0.58233	0.84989	-0.235
389.2482_7.74	0.07458	0.17974	0.65841	-0.603	0.093653	0.21455	0.63186	-0.662
277.2174_1.01	1.59E-05	0.005019	0.22812	-2.132	0.030674	0.14993	0.58642	-0.77
295.2271_6.49	0.000238	0.022032	0.30588	-1.709	0.022965	0.11938	0.5356	-0.901
337.2754_1.64	0.011403	0.069587	0.45535	-1.135	0.2775	0.49867	0.81581	-0.294
301.2518_6.25	0.87686	0.91242	1.124	0.1686	0.43556	0.56736	1.2368	0.3066
353.2319_4.92	0.12958	0.23399	0.51964	-0.944	0.087285	0.18436	0.4818	-1.054
253.2165_7.97	0.001945	0.035667	0.53129	-0.912	0.010505	0.087266	0.5436	-0.879
271.227_7.77	0.020821	0.084081	3.374	1.7544	0.017268	0.10968	3.8072	1.9287
137.1328_7.7	0.000406	0.018141	0.4155	-1.267	0.001316	0.067478	0.45025	-1.151
178.0501_0.67	0.010548	0.062803	0.40718	-1.296	0.006056	0.086891	0.37903	-1.4
299.2582_7.18	0.022214	0.086922	2.3285	1.2194	0.016919	0.10889	2.0011	1.0008
297.2426_7.36	0.040647	0.12063	2.5607	1.3565	0.026598	0.12548	2.3047	1.2046
373.2352_6.94	0.010168	0.048983	0.66422	-0.59	0.7949	0.85188	1.0314	0.0447
321.2412_1.31	0.10398	0.26421	2.4186	1.2742	0.060682	0.20931	2.0683	1.0484
293.2113_6.12	0.000219	0.022032	0.23385	-2.096	0.009623	0.086891	0.41732	-1.261
351.2093_0.78	0.78233	0.94454	1.3029	0.3817	0.92128	0.97778	1.132	0.1789
201.1236_0.97	0.51265	0.65829	0.53399	-0.905	0.50721	0.63801	0.62184	-0.685
187.1077_0.99	0.22934	0.39088	0.346	-1.531	0.33406	0.48525	0.46502	-1.105
305.2481_8.05	0.001026	0.020245	3.0895	1.6274	0.003462	0.067478	2.5468	1.3487
365.1867_0.64	0.17265	0.67917	1.2297	0.2983	0.19852	0.66011	1.317	0.3972
421.2346_0.81	0.33353	0.74661	0.90307	-0.147	0.83103	0.96517	1.0014	0.0021
131.0707_2.5	0.39317	0.55439	0.39194	-1.351	0.079016	0.19672	0.17412	-2.522
329.2318_4.29	0.065777	0.14856	0.57732	-0.793	0.018068	0.086304	0.492	-1.023
465.3038_10.65	0.25792	0.42131	2.5653	1.3591	0.021675	0.11753	3.8268	1.9361
373.2116_7.93	0.065474	0.14822	1.6235	0.6991	0.42584	0.55803	1.185	0.2449
369.228_4.29	0.72999	0.79856	0.92894	-0.106	0.092699	0.1908	0.69652	-0.522
201.1125_3.76	0.023484	0.088753	0.42617	-1.231	0.027652	0.12658	0.46136	-1.116
391.2846_5.41	0.5931	0.72696	1.212	0.2774	0.3006	0.45321	1.2421	0.3128
305.2478_8.5	0.004487	0.046388	3.9177	1.97	0.006699	0.086891	3.5515	1.8284
514.2838_4.26	0.25723	0.42079	0.081169	-3.623	0.44305	0.58546	0.1063	-3.234
193.0498_2.87	0.025585	0.093812	0.30837	-1.697	0.006243	0.086891	0.20059	-2.318
269.0448_4.04	0.99124	0.99586	1.0025	0.0036	0.18209	0.32743	0.96499	-0.051
195.0044_11.59	0.038391	0.10376	1.3852	0.4701	0.27355	0.40228	1.3472	0.43
375.2521_1.32	0.001486	0.016327	0.33328	-1.585	0.12357	0.31169	0.68518	-0.545
253.0496_3.58	0.45175	0.60382	0.60279	-0.73	0.25034	0.40218	0.33362	-1.584
377.085_0.42	0.80885	0.89891	2.0781	1.0553	0.001397	0.086891	0.15034	-2.734
546.3547_1.73	0.23544	0.45805	3.0385	1.6034	0.035845	0.15996	5.543	2.4707
522.3567_1.29	0.51422	0.72097	1.3	0.3785	0.29937	0.52063	1.7423	0.801
452.2777_6.69	0.97721	0.98779	1.3839	0.4688	0.16939	0.31322	2.2471	1.1681
480.3072_7.45	0.22578	0.38819	3.275	1.7115	0.003033	0.086891	5.3956	2.4318
483.273_7.29	0.15419	0.29667	1.4791	0.5647	0.090088	0.20975	1.697	0.763
376.2578_1.32	0.32889	0.55779	0.792	-0.336	0.62276	0.81082	0.89768	-0.156
303.2302_2.2	0.005913	0.045887	0.50625	-0.982	0.011986	0.10653	0.54101	-0.886
165.0549_2.81	0.11919	0.24807	0.47183	-1.084	0.057246	0.16685	0.35242	-1.505
509.2879_7.55	0.078254	0.1852	1.7684	0.8224	0.018203	0.11118	1.7256	0.7871
323.256_7.19	0.013259	0.054929	2.4266	1.2789	0.009975	0.074222	2.0686	1.0487
377.2676_1.65	9.84E-05	0.005019	0.25968	-1.945	0.29969	0.52063	1.0108	0.0156
164.071_0.73	0.0607	0.15794	0.38388	-1.381	0.13857	0.2716	0.5564	-0.846
165.055_0.49	0.22263	0.34178	0.51739	-0.951	0.23703	0.3616	0.59869	-0.74
295.228_1.01	8.92E-05	0.005019	0.23954	-2.062	0.082866	0.25155	0.69966	-0.515
492.2131_5.94	0.52603	0.67269	1.0628	0.0879	0.52424	0.65296	0.87636	-0.19
193.0693_2.57	0.011929	0.051979	0.43171	-1.212	0.010764	0.075462	0.43321	-1.207
203.0819_1.22	0.44216	0.59796	0.42462	-1.236	0.28697	0.43974	0.36604	-1.45
591.3182_0.63	0.012252	0.072299	2.1538	1.1069	0.067015	0.22152	2.3424	1.228
391.2848_9.1	0.00414	0.032544	1.4046	0.4902	0.001308	0.067478	1.3451	0.4277

	08. Vehicle_SD_14_vs_Vehicle_Pre_1				09. Bio300 OS SD_14_vs_Vehicle_SD_14				10. Bio300 OP SD_14_vs_Vehicle_SD_14			
**mz_RT**	**p-value**	**FDR**	**FC**	**LOG2FC**	**p-value**	**FDR**	**FC**	**LOG2FC**	**p-value**	**FDR**	**FC**	**LOG2FC**
830.5883_9.98	0.53646	0.63515	0.82045	-0.286	0.044508	0.1479	0.25509	-1.971	0.016355	0.048171	0.18571	-2.429
140.0712_0.57	0.00327	0.014151	0.56819	-0.816	0.15829	0.26655	1.3497	0.4327	0.013981	0.031527	1.6019	0.6798
321.2404_7.36	0.00047	0.00489	10.998	3.4592	0.49768	0.60642	0.62337	-0.682	0.32426	0.39419	0.4293	-1.22
279.2331_0.97	0.24713	0.3601	1.2183	0.2848	0.092888	0.19587	1.6975	0.7634	0.04182	0.072652	1.844	0.8828
389.269_5.94	0.014769	0.040511	0.61038	-0.712	0.4246	0.52927	1.2442	0.3152	0.22343	0.28674	1.2449	0.3161
389.2482_7.74	0.000458	0.007047	0.30684	-1.704	0.63835	0.71421	0.99271	-0.011	0.32613	0.38698	1.2764	0.3521
277.2174_1.01	0.38619	0.50134	1.5668	0.6478	0.21516	0.32206	0.94928	-0.075	0.11948	0.1734	1.1257	0.1708
295.2271_6.49	0.2894	0.39316	1.2431	0.314	0.16828	0.28369	1.1289	0.1749	0.13768	0.19829	1.1498	0.2014
337.2754_1.64	5.76E-05	0.001476	0.13161	-2.926	0.41878	0.53528	1.1699	0.2264	0.28808	0.36277	1.4986	0.5836
301.2518_6.25	0.000291	0.004038	10.828	3.4366	0.55952	0.66528	0.9494	-0.075	0.17258	0.23709	0.42423	-1.237
353.2319_4.92	0.43505	0.5355	0.99705	-0.004	0.53926	0.64756	1.2983	0.3766	0.007108	0.027044	1.8996	0.9257
253.2165_7.97	0.023757	0.054411	0.66086	-0.598	0.098453	0.20588	1.3069	0.3861	0.094518	0.14885	1.4078	0.4934
271.227_7.77	0.002654	0.016028	17.296	4.1124	0.012519	0.12317	0.2794	-1.84	0.000169	0.007142	0.016292	-5.94
137.1328_7.7	0.002298	0.01203	0.52999	-0.916	0.02235	0.12346	1.586	0.6654	0.006559	0.02602	1.7505	0.8078
178.0501_0.67	0.000109	0.003303	0.3007	-1.734	0.028987	0.13857	3.6933	1.8849	0.000767	0.015898	2.9098	1.5409
299.2582_7.18	8.11E-05	0.003123	7.4649	2.9001	0.20644	0.32405	0.66132	-0.597	0.045591	0.090013	0.4107	-1.284
297.2426_7.36	0.000291	0.005509	10.657	3.4137	0.59638	0.68067	0.83498	-0.26	0.29726	0.35784	0.45209	-1.145
373.2352_6.94	5.93E-05	0.001947	0.29695	-1.752	0.073291	0.17712	1.5663	0.6474	0.011328	0.035478	1.9318	0.95
321.2412_1.31	0.000225	0.002588	10.892	3.4453	0.85476	0.90014	0.90267	-0.148	0.86085	0.89729	0.67559	-0.566
293.2113_6.12	0.042949	0.084977	0.58015	-0.786	0.035925	0.14032	2.3595	1.2385	0.016074	0.047736	2.3743	1.2475
351.2093_0.78	0.041379	0.21622	2.1407	1.0981	0.50361	0.79558	1.0387	0.0548	0.84378	0.93941	1.0876	0.1211
201.1236_0.97	0.000566	0.007774	0.26997	-1.889	0.063242	0.16695	2.1866	1.1287	0.002529	0.022521	2.7844	1.4774
187.1077_0.99	0.000898	0.009535	0.25333	-1.981	0.019862	0.12884	2.8364	1.504	0.005984	0.031489	2.9644	1.5677
305.2481_8.05	0.29794	0.39507	1.1259	0.1711	0.51184	0.61924	0.92342	-0.115	0.04432	0.084447	0.73335	-0.447
365.1867_0.64	0.014529	0.11611	2.0112	1.0081	0.21944	0.59969	0.68961	-0.536	0.03063	0.19056	0.54914	-0.865
421.2346_0.81	0.005567	0.07162	1.2811	0.3574	0.48043	0.78235	1.0797	0.1107	0.52692	0.81791	1.0468	0.066
131.0707_2.5	0.26719	0.36833	0.95113	-0.072	0.44461	0.54964	1.4897	0.5751	0.001508	0.019826	4.2388	2.0837
329.2318_4.29	0.000513	0.005169	0.3765	-1.409	0.02234	0.12346	2.6385	1.3997	0.000717	0.010401	3.0284	1.5986
465.3038_10.65	0.39604	0.50145	2.6208	1.39	0.079846	0.18506	0.79458	-0.332	0.003387	0.025294	0.084116	-3.572
373.2116_7.93	0.008914	0.026085	0.56815	-0.816	0.009307	0.10071	1.8402	0.8799	0.006886	0.026679	1.74	0.7991
369.228_4.29	0.001579	0.009763	0.5416	-0.885	0.1381	0.24952	1.59	0.669	0.015411	0.042151	1.7253	0.7869
201.1125_3.76	0.043295	0.085559	0.606	-0.723	0.044253	0.1479	2.0803	1.0568	0.009687	0.036969	2.3843	1.2535
391.2846_5.41	0.57602	0.67166	1.2677	0.3422	0.12081	0.23214	1.453	0.539	0.036438	0.0774	1.6285	0.7035
305.2478_8.5	0.002499	0.015617	4.7105	2.2359	0.1031	0.2105	0.55943	-0.838	0.004847	0.029088	0.25182	-1.99
514.2838_4.26	0.021474	0.051327	0.55113	-0.86	0.41968	0.52606	1.3926	0.4778	0.014809	0.046006	1.7373	0.7969
193.0498_2.87	0.00116	0.010419	0.12885	-2.956	0.019824	0.12884	4.7538	2.2491	0.001318	0.018479	4.8823	2.2876
269.0448_4.04	0.095978	0.15979	0.56333	-0.828	0.89845	0.91891	0.8853	-0.176	0.24544	0.30909	1.3443	0.4269
195.0044_11.59	0.003446	0.014675	2.3145	1.2107	0.027183	0.12941	0.54387	-0.879	0.031676	0.068011	0.55112	-0.86
375.2521_1.32	1.64E-05	0.000974	0.1312	-2.93	0.14346	0.25142	1.7323	0.7927	0.020723	0.041916	2.8261	1.4988
253.0496_3.58	0.006769	0.025513	0.24466	-2.031	0.2707	0.38986	1.3766	0.4611	0.025603	0.060904	2.175	1.121
377.085_0.42	0.008939	0.029765	0.24279	-2.042	0.15846	0.27579	2.7062	1.4363	0.14889	0.20913	1.4783	0.5639
546.3547_1.73	0.058545	0.12294	0.17593	-2.507	0.64623	0.73954	1.4715	0.5573	0.016757	0.035462	0.19171	-2.383
522.3567_1.29	0.000393	0.003409	0.15276	-2.711	0.76684	0.84626	1.2033	0.267	0.49625	0.57333	0.70791	-0.498
452.2777_6.69	0.32962	0.4328	1.9635	0.9734	0.060083	0.16358	0.34501	-1.535	0.006172	0.031987	0.21912	-2.19
480.3072_7.45	0.9079	0.93522	1.1537	0.2063	0.036772	0.14139	0.35374	-1.499	0.000474	0.012175	0.10364	-3.27
483.273_7.29	0.85418	0.89876	1.0756	0.1051	0.033799	0.1393	0.4593	-1.123	0.031968	0.069549	0.46707	-1.098
376.2578_1.32	2.05E-07	0.000146	0.26234	-1.931	0.26093	0.37336	1.246	0.3173	0.060591	0.099912	1.7526	0.8095
303.2302_2.2	0.000672	0.004617	0.44339	-1.173	0.072851	0.17397	1.9335	0.9513	0.023069	0.044759	1.8781	0.9093
165.0549_2.81	0.009943	0.031844	0.44805	-1.158	0.058553	0.16247	2.2305	1.1574	0.008661	0.036172	2.825	1.4982
509.2879_7.55	0.22873	0.32422	1.7718	0.8253	0.16977	0.28484	0.5351	-0.902	0.34593	0.40696	0.63017	-0.666
323.256_7.19	6.12E-05	0.001947	8.7622	3.1313	0.11603	0.22576	0.49834	-1.005	0.043198	0.082939	0.38503	-1.377
377.2676_1.65	5.01E-05	0.001476	0.095989	-3.381	0.038906	0.1292	2.3922	1.2584	0.019562	0.040251	2.9896	1.5799
164.071_0.73	0.007083	0.026373	0.32366	-1.628	0.009902	0.12199	2.9403	1.556	0.001623	0.020229	3.4169	1.7727
165.055_0.49	0.017212	0.041184	0.45454	-1.138	0.030499	0.13115	2.0867	1.0612	0.000937	0.010657	2.5442	1.3472
295.228_1.01	0.55989	0.67756	2.1658	1.1149	0.2593	0.37251	0.8049	-0.313	0.13883	0.19785	0.9798	-0.029
492.2131_5.94	0.37641	0.48162	0.96862	-0.046	0.086253	0.19231	2.494	1.3185	0.014311	0.045243	3.0706	1.6185
193.0693_2.57	0.001032	0.007403	0.30301	-1.723	0.01786	0.11798	2.3394	1.2261	0.006504	0.025839	2.8754	1.5238
203.0819_1.22	0.002187	0.014554	0.16508	-2.599	0.010503	0.12227	3.727	1.898	4.54E-05	0.003973	3.8383	1.9405
591.3182_0.63	0.000112	0.001819	31.171	4.9621	0.00442	0.048271	0.10303	-3.279	0.00101	0.010028	0.063129	-3.986
391.2848_9.1	0.04027	0.074799	1.7344	0.7944	0.059895	0.1623	0.59296	-0.754	0.032268	0.068909	0.56075	-0.835

Additional file 1. Table 4: The Mummichog (version 2.06) pathway analysis outcome for side effect

Pathway	Bio300 OS Pre_7 vs. Vehicle_Pre_7		Bio300 OP Pre_7 vs. Vehicle_Pre_7		Bio300 OS Pre_1 vs. Vehicle_Pre_1		Bio300 OP Pre_1 vs. Vehicle_Pre_1	
	overlap size	*p-value*	*overlap size*	*p-value*	*overlap size*	*p-value*	*overlap size*	*p-value*
**De novo fatty acid biosynthesis**	-	-	-	-	12(30)	0.030334	-	-
**Linoleate metabolism**	-	-	6(21)	0.001176	18(34)	5.04E-04	-	-
**C21-steroid hormone biosynthesis and metabolism**	-	-	4(22)	0.031846	-	-	-	-
**Vitamin A (retinol) metabolism**	-	-	3(19)	0.043946	9(19)	0.017141	5(19)	0.03882
**Valine, leucine and isoleucine degradation**	4(11)	7.56E-04	-	-	-	-	-	-
**Omega-3 fatty acid metabolism**	-	-	-	-	8(11)	0.001092	-	-
**Hexose phosphorylation**	2(6)	0.010587	-	-	-	-	-	-
**Glycerophospholipid metabolism**	3(14)	0.009495	-	-	-	-	3(14)	0.037476
**Leukotriene metabolism**	-	-	-	-	6(12)	0.001933	-	-
**Pentose phosphate pathway**	2(8)	0.01815	-	-	-	-	-	-
**Bile acid biosynthesis**	-	-	-	-	-	-	17(72)	0.003109
**Pyruvate Metabolism**	1(5)	0.048819	-	-	-	-	-	-
**Glycolysis and Gluconeogenesis**	2(5)	0.006554	-	-	-	-	-	-
**Vitamin D3 (cholecalciferol) metabolism**	-	-	-	-	-	-	9(29)	0.004201
**Propanoate metabolism**	2(4)	0.004285	-	-	-	-	-	-
**Fructose and mannose metabolism**	2(4)	0.004285	-	-	-	-	-	-
**Limonene and pinene degradation**	-	-	-	-	4(5)	0.010419	-	-
**Arachidonic acid metabolism**	-	-	4(26)	0.026384	12(26)	0.010167	-	-
**Ubiquinone Biosynthesis**	1(4)	0.044282	-	-	-	-	-	-
**Glycine, serine, alanine and threonine metabolism**	2(17)	0.011176	-	-	-	-	-	-
**Urea cycle/amino group metabolism**	2(22)	0.018318	-	-	-	-	-	-
**Putative anti-Inflammatory metabolites formation from EPA**	-	-	-	-	3(5)	0.01294	-	-
**Starch and Sucrose Metabolism**	1(2)	0.028821	-	-	-	-	-	-
**N-Glycan biosynthesis**	1(1)	0.017646	-	-	-	-	-	-
**Galactose metabolism**	2(8)	0.01815	-	-	-	-	-	-
**Vitamin B5 - CoA biosynthesis from pantothenate**	-	-	1(1)	0.022267	-	-	-	-
**CoA Catabolism**	-	-	1(1)	0.022267	-	-	-	-
**Alanine and Aspartate Metabolism**	-	-	-	-	3(6)	0.025796	-	-
**Chondroitin sulfate degradation**	1(2)	0.028821	-	-	-	-	-	-
**Heparan sulfate degradation**	1(2)	0.028821	-	-	-	-	-	-
**Vitamin H (biotin) metabolism**	-	-	-	-	3(4)	0.029157	-	-
**Porphyrin metabolism**	-	-	-	-	3(4)	0.029157	2(4)	0.03882

**Lysine metabolism**	-	-	-	-	2(3)	0.035963	-	-
**Keratan sulfate degradation**	1(3)	0.037056	-	-	-	-	-	-
**Beta-Alanine metabolism**	1(4)	0.044282	-	-	-	-	-	-
**Vitamin B9 (folate) metabolism**	-	-	-	-	2(2)	0.033611	-	-
**Omega-6 fatty acid metabolism**	-	-	-	-	-	-	1(1)	0.034199
**Glycosphingolipid biosynthesis - ganglioseries**	1(3)	0.037056	-	-	-	-	-	-
**Glycosphingolipid biosynthesis - globoseries**	1(3)	0.037056	-	-	-	-	-	-
**N-Glycan Degradation**	1(3)	0.037056	-	-	-	-	-	-
**Butanoate metabolism**	1(6)	0.037644	-	-	-	-	-	-
**Methionine and cysteine metabolism**	-	-	-	-	5(10)	0.039997	-	-
**Phosphatidylinositol phosphate metabolism**	1(4)	0.044282	-	-	-	-	-	-
**Squalene and cholesterol biosynthesis**	1(5)	0.048819	-	-	-	-	-	-

Additional file 1. Table 5: The Mummichog pathway analysis outcome for radiation effect and BIO 300 protective effect at time SD 3.

Pathway	Vehicle_SD_3 vs. Vehicle_Pre_1		Bio300 OS SD_3 vs. Vehicle_SD_3		Bio300 OP SD_3 vs. Vehicle_SD_3	
	overlap size	***p-value***	***overlap size***	***p-value***	***overlap size***	***p-value***
**De novo fatty acid biosynthesis**	-	-	25(30)	8.40E-05	-	-
**Linoleate metabolism**	-	-	28(34)	8.40E-05	25(34)	5.04E-04
**C21-steroid hormone biosynthesis and metabolism**	-	-	36(46)	8.40E-05	15(22)	0.00193261
**Vitamin A (retinol) metabolism**	-	-	16(19)	2.52E-04	14(19)	0.00470549
**Fatty acid activation**	-	-	20(26)	6.72E-04	-	-
**Omega-3 fatty acid metabolism**	-	-	3(3)	0.01688934	9(11)	0.00638602
**Hexose phosphorylation**	-	-	-	-	6(6)	0.00151248
**Glycerophospholipid metabolism**	11(14)	0.00277288	-	-	10(14)	0.00462146
**Leukotriene metabolism**	8(12)	0.03713974	-	-	-	-
**Pentose phosphate pathway**	-	-	-	-	7(8)	0.0025208
**Prostaglandin formation from arachidonate**	11(15)	0.00638602	17(23)	0.00319301	-	-
**Pyruvate Metabolism**	-	-	-	-	5(5)	0.00352912
**Glycolysis and Gluconeogenesis**	-	-	-	-	5(5)	0.00352912
**Fructose and mannose metabolism**	-	-	-	-	4(4)	0.00848668
**Vitamin B3 (nicotinate and nicotinamide) metabolism**	5(5)	0.00504159	-	-	-	-
**Tyrosine metabolism**	19(31)	0.00999916	-	-	-	-
**Limonene and pinene degradation**	-	-	5(5)	0.00974708	5(5)	0.00739434
**Arachidonic acid metabolism**	11(16)	0.0110915	18(26)	0.0082346	16(26)	0.02865305
**Ubiquinone Biosynthesis**	-	-	-	-	4(4)	0.00848668
**Androgen and estrogen biosynthesis and metabolism**	8(11)	0.02159482	9(11)	0.00932695	4(5)	0.03419881
**Carnitine shuttle**	12(17)	0.00932695	-	-	-	-
**Glycine, serine, alanine and threonine metabolism**	8(12)	0.03713974	-	-	-	-
**Fatty Acid Metabolism**	-	-	12(18)	0.03453491	-	-
**Saturated fatty acids beta-oxidation**	-	-	4(4)	0.02050248	-	-
**Di-unsaturated fatty acid beta-oxidation**	-	-	4(4)	0.02050248	-	-
**Chondroitin sulfate degradation**	3(3)	0.03176204	-	-	-	-
**Heparan sulfate degradation**	3(3)	0.03176204	-	-	-	-
**Lysine metabolism**	3(3)	0.03016553	-	-	-	-
**Prostaglandin formation from dihomo gama-linoleic acid**	3(3)	0.03016553	-	-	-	-
**Glycosphingolipid metabolism**	-	-	-	-	11(17)	0.03713974

Additional file 1. Table 6: The pathway analysis outcome for time SD 14 radiation effect and BIO 300 protective effect

	Vehicle_SD_14 vs. Vehicle_Pre_1		Bio300 OS SD_14 vs. Vehicle_SD_14		Bio300 OP SD_14 vs. Vehicle_SD_14	
**Pathway**	***overlap size***	***p-value***	***overlap size***	***p-value***	***overlap size***	***p-value***
**De novo fatty acid biosynthesis**	-	-	6(10)	0.037308	8(10)	0.04403
**C21-steroid hormone biosynthesis and metabolism**	-	-	-	-	18(22)	0.003277
**Vitamin A (retinol) metabolism**	-	-	4(6)	0.04924	14(19)	0.034367
**Fatty acid activation**	-	-	5(8)	0.044282	7(8)	0.023443
**Hexose phosphorylation**	6(6)	0.009159	-	-	-	-
**Glycerophospholipid metabolism**	13(14)	0.001597	-	-	11(14)	0.026216
**Bile acid biosynthesis**	-	-	35(72)	0.014032	-	-
**Vitamin D3 (cholecalciferol) metabolism**	-	-	18(29)	0.003613	22(29)	0.008319
**Fructose and mannose metabolism**	4(4)	0.036888	4(5)	0.031678	-	-
**Tyrosine metabolism**	21(28)	0.010251	17(28)	0.005462	22(31)	0.02546
**Limonene and pinene degradation**	-	-	4(5)	0.031678	5(5)	0.019074
**Ubiquinone Biosynthesis**	4(4)	0.036888	-	-	-	-
**Biopterin metabolism**	-	-	4(4)	0.009327	4(4)	0.033106
**Urea cycle/amino group metabolism**	-	-	12(22)	0.035627	17(22)	0.012016
**Fatty Acid Metabolism**	-	-	3(3)	0.015965	-	-
**Starch and Sucrose Metabolism**	5(5)	0.017057	-	-	-	-
**Di-unsaturated fatty acid beta-oxidation**	4(4)	0.030838	-	-	-	-
**Vitamin H (biotin) metabolism**	4(4)	0.030838	-	-	-	-
**Porphyrin metabolism**	4(4)	0.030838	-	-	-	-
**Beta-Alanine metabolism**	-	-	-	-	4(4)	0.033106
